# Dual-Defect Engineering Strategy Enables High-Durability Rechargeable Magnesium-Metal Batteries

**DOI:** 10.1007/s40820-024-01410-8

**Published:** 2024-04-29

**Authors:** Fuyu Chen, Bai-Qing Zhao, Kaifeng Huang, Xiu-Fen Ma, Hong-Yi Li, Xie Zhang, Jiang Diao, Jili Yue, Guangsheng Huang, Jingfeng Wang, Fusheng Pan

**Affiliations:** 1 National Innovation Center for Lndustry-Education Integration of Energy Storage Technology, School of Materials Science and Engineering, Chongqing University, Chongqing, 400044 People’s Republic of China; 2 National Magnesium Alloy Material Engineering Technology Research Center, Chongqing University, Chongqing, 400044 People’s Republic of China; 3 Materials and Energy Division, Beijing Computational Science Research Center, Beijing, 100193 People’s Republic of China; 4 School of Materials Science and Engineering, Northwestern Polytechnical University, Xi’an, 710072 People’s Republic of China; 5 National Key Laboratory of Advanced Casting Technologies, Chongqing University, Chongqing, 400044 People’s Republic of China

**Keywords:** Rechargeable magnesium-metal batteries, Dual-defect engineering, Vanadium-based cathode, High durability, Lamellar structure

## Abstract

Rechargeable magnesium-metal batteries (RMMBs) are promising next-generation secondary batteries; however, their development is inhibited by the low capacity and short cycle lifespan of cathodes. Although various strategies have been devised to enhance the Mg^2+^ migration kinetics and structural stability of cathodes, they fail to improve electronic conductivity, rendering the cathodes incompatible with magnesium-metal anodes. Herein, we propose a dual-defect engineering strategy, namely, the incorporation of Mg^2+^ pre-intercalation defect (P-Mg_d_) and oxygen defect (O_d_), to simultaneously improve the Mg^2+^ migration kinetics, structural stability, and electronic conductivity of the cathodes of RMMBs. Using lamellar V_2_O_5_·nH_2_O as a demo cathode material, we prepare a cathode comprising Mg_0.07_V_2_O_5_·1.4H_2_O nanobelts composited with reduced graphene oxide (MVOH/rGO) with P-Mg_d_ and O_d_. The O_d_ enlarges interlayer spacing, accelerates Mg^2+^ migration kinetics, and prevents structural collapse, while the P-Mg_d_ stabilizes the lamellar structure and increases electronic conductivity. Consequently, the MVOH/rGO cathode exhibits a high capacity of 197 mAh g^−1^, and the developed Mg foil//MVOH/rGO full cell demonstrates an incredible lifespan of 850 cycles at 0.1 A g^−1^, capable of powering a light-emitting diode. The proposed dual-defect engineering strategy provides new insights into developing high-durability, high-capacity cathodes, advancing the practical application of RMMBs, and other new secondary batteries.

**Supplementary Information:**

The online version contains supplementary material available at 10.1007/s40820-024-01410-8.

## Highlights

- MVOH/rGO cathode with dual defect of Mg^2+^ pre-intercalation defect (P-Mg_d_) and surface oxygen defect (O_d_) is prepared.
- The dual defect of O_d_ and P-Mg_d_ in MVOH/rGO lamellar structure effectively enhances Mg^2+^ migration kinetics, structural stability, and electronic conductivity.
- The Mg foil//MVOH/rGO full cell achieves an ultralong lifespan of 850 cycles at 0.1 A g^−1^ and powers an orange light-emitting diode.

**Supplementary Information:**

The online version contains supplementary material available at 10.1007/s40820-024-01410-8.

## Graphical abstract

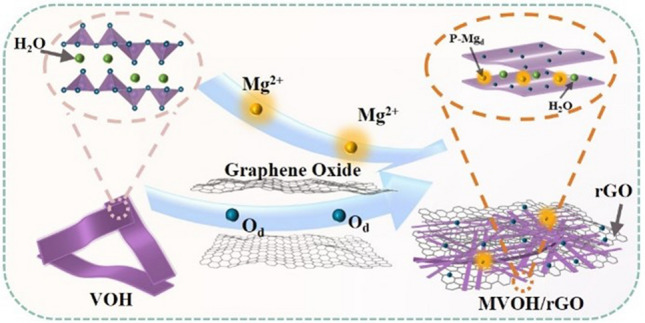

## Introduction

Rechargeable magnesium-metal batteries (RMMBs) have emerged as promising next-generation energy-storage devices, surpassing lithium-ion batteries (LIBs) due to their high theoretical volumetric capacity (3833 mAh cm^−3^) and natural abundance (ranked 3rd in seawater and 8th in the earth’s crust) as well as the lower redox potential (− 2.37 V vs. standard hydrogen electrode) of the magnesium-metal anodes [1–4]. Furthermore, the absence of sharp dendrites in magnesium-metal anodes ensures the safety of RMMBs over LIBs [5, 7]. However, the bivalent nature of Mg^2+^ and resulting high positive charge density lead to strong interactions between the inserted Mg^2+^ and host materials, resulting in sluggish diffusion kinetics of Mg^2+^ in cathodes and thus leading to the low capacity of RMMB cathodes [8, 9]. In addition, due to the strong interactions, the repetitive insertion and extraction of Mg^2+^ usually cause the collapse of the host materials, resulting in a short cycle lifespan of the cathodes and RMMBs [10, 11].

Present efforts focus on improving the Mg^2+^ diffusion kinetics and cycle stability of lamellar structures as RMMB cathodes because lamellar structures are believed to exhibit limited structural variation during the insertion/extraction of Mg^2+^ [12, 14]. Vanadium-based cathodes have attracted wide attention because of their tunable lamellar structures and high theoretical charge-storage capacity [15, 17]. Metal ions, including Mg^2+^ [5, 18, 19], Mn^2+^ [20], Co^2+^, and Ni^2+^ [21], or organic molecules [10, 22, 23], such as alkylamines, have been pre-intercalated into interlayers of vanadium-based lamellar structures to enlarge the interlayer spacing for favorable Mg^2+^ diffusion paths, which can lead to the high capacity of cathodes for Mg^2+^ storage. These pre-intercalated cations or molecules also function as pillars to stabilize the lamellar structure, increasing the cycle lifespan of these lamellar structure cathodes [24, 27]. However, strong interactions between Mg^2+^ and host lamellar structures still exist, resulting in a limited improvement of the cathode cycle lifespan. Furthermore, these efforts only increase the ion conductivity of vanadium-based cathodes; however, the electronic conductivity of these cathodes is low, resulting in incompatibility between slow Mg^2+^ insertion/extraction in the cathodes and fast dissolution/deposition of magnesium-metal anodes [28, 31]. Consequently, most vanadium-based cathodes can only be assembled into magnesium-ion batteries instead of RMMBs, which forfeits the advantages of magnesium-metal anodes. Therefore, to develop RMMB cathodes with high capacity and cycle stability, the steric hindrance of Mg^2+^ diffusion and the interaction between Mg^2+^ and host structures must be simultaneously decreased, while also increasing the electronic conductivity of the cathodes. At present, the dual-defect engineering includes the combination of ion/molecule pre-intercalation with oxygen vacancies or dual ions co-insertion, and the coexistence of cationic and anionic defects, etc. [32, 34]. For instance, the synergistic construction of Cr^3+^ pre-intercalation and oxygen defects in vanadium oxide hydrate can effectively boost the electrical conductivity and ion migration kinetics of electrode [32]. Furthermore, the V/O dual defects in NH_4_V_4_O_10_ materials are reported to effectively lower the migration energy barrier, facilitating ion transport in the cathode material [34]. The present works demonstrate the dual-defect engineering to be a powerful strategy to promote the ion migration kinetics and electrical conductivity of cathode materials.

Herein, we propose a dual-defect engineering strategy, namely, the incorporation of interlayer Mg^2+^ pre-intercalation defect (P-Mg_d_) and surface oxygen defect (O_d_), to synergistically enhance the Mg^2+^ diffusion kinetics, structural stability, and electronic conductivity of RMMB cathodes. We utilized a typical lamellar compound of hydrated vanadium pentoxide (V_2_O_5_·nH_2_O) as a demo cathode material and prepared a dual-defect cathode comprising Mg_0.07_V_2_O_5_·1.4H_2_O nanobelts composited with reduced graphene oxide (MVOH/rGO). The O_d_ weakens the interaction between pre-intercalated Mg^2+^ and V–O layers in V_2_O_5_·nH_2_O, enlarging the interlayer spacing of V_2_O_5_·nH_2_O and the P-Mg_d_ causes the lamellar structure to have a larger spacing, facilitating the reversible diffusion kinetics of Mg^2+^ in the MVOH/rGO cathode. In addition, the pinning effect of the P-Mg_d_ stabilizes the lamellar structure, leading to a long cycle life of the MVOH/rGO cathode. Furthermore, the interaction between Mg^2+^ and the host structure, alleviated by the O_d_, avoids structural collapse due to the limited lattice distortion, further prolonging the cathode cycle lifespan. Furthermore, the P-Mg_d_ causes the overlap of the conduction and valence bands of V_2_O_5_·nH_2_O, increasing the electronic conductivity of V_2_O_5_·nH_2_O, and the O_d_ generates hopping paths for *3d* electrons from V^4+^ to V^5+^ in V_2_O_5_·nH_2_O, connecting with the conductive network of the rGO substrate, substantially increasing the electronic conductivity of the MVOH/rGO cathode. Consequently, the MVOH/rGO cathode exhibits a high capacity of 197 mAh g^−1^ at 0.02 A g^−1^, a superior rate performance of 64 mAh g^−1^ at 3.0 A g^−1^, and an ultralong cycle life of 7000 cycles. With Mg metal as the anode, the developed Mg foil//MVOH/rGO full cell can power an orange light-emitting diode (LED), retaining 84% of the initial capacity after 850 cycles at 0.1 A g^−1^, surpassing the lifespan of most reported RMMBs. The proposed dual-defect engineering strategy sheds light on a new avenue for developing high-durability, high-capacity cathodes, which will promote the practical application of RMMBs and other new secondary batteries.

## Experimental Section

### Materials

Vanadium pentoxide (purity > 99%) and 30% hydrogen peroxide were purchased from ChuanDong Chemicals Co. Magnesium nitrate hexahydrate (purity > 99%) and acetonitrile (AR > 98%) were purchased from Aladdin Co. CTAB (purity > 99%), while N-methyl pyrrolidone and polyvinylidene fluoride were purchased from Shanghai Maclin Biochemical Technology Co. The APC and Mg(TFSI)_2_ electrolyte were purchased from Suzhou Duoduo Chemical Technology Co. All reagents and solvents are used directly without any treatment.

### Preparation of Mg_0.07_V_2_O_5_·1.4H_2_O/rGO Nanobelts

In detail, 2-mmol V_2_O_5_ powder was dissolved in 50 mL of deionized water and continuously stirred for 30 min at 25 °C. Then, 8-mL 30% hydrogen peroxide was added to the V_2_O_5_ aqueous solution stirring continuously for 60 min to remove the bubbles. Afterward, 0.1-mmol magnesium nitrate was dissolved in the above homogeneous solution, followed by adding 35-mL 2.59 mg mL^−1^ GO aqueous solution and placing it in an ice bath ultrasound for 60 min. After being thoroughly mixed, it was then placed in a blast oven at 190 °C for 12 h. Subsequently, the as-prepared Mg_0.07_V_2_O_5_·1.4H_2_O/rGO was freeze-dried for 60 h. The GO aqueous solution was synthesized by the previous report work [35].

### Characterizations

The surface morphology and microstructure of the as-prepared material were characterized by environmental scanning electron microscopy (ESEM, Thermo Fisher) and field emission transmission electron microscopy (FETEM, Talos). Elemental valence state and chemical bond compositions were characterized by X-ray photoelectron spectrometry (XPS, Thermo Fisher ESCALAB) with Al-K*α* radiation as the excitation source. The internal elemental valence and chemical information were characterized by XPS after Ar ion etching, where the etched spot size and voltage were 1.5 mm and 3000 eV, respectively. X-ray diffractometer (XRD, Panalytical) with Cu-K*α* radiation (*λ* = 0.15406 nm) was used to test the phase composition of the samples. Thermogravimetric analyzer (TGA, Mettler Toledo) was conducted to calculate the mass of structural water. The functional groups of graphene oxide and vanadium oxide were confirmed by Raman spectroscopy (HORIBA).

### Electrochemical Measurements

The active material, acetylene black conductor, and polyvinylidene fluoride binder were ground with the mass ratio of 7:2:1 as a homogeneous slurry, which was then coated on the carbon paper collector and vacuum dried at 80 °C for 12 h. Subsequently, the dried slurry was cut into disks with a diameter of 12 mm to obtain the electrodes, with a loading mass of 1.5–2.0 mg. Mg foils were cut into disks with a diameter of 14 mm and polished by metallographic sandpaper (800 mesh) to remove the magnesium surface passivation layer. 0.4 M APC-CTAB electrolyte was synthesized with the reported literature [23]. Half-cells were assembled by active carbon (AC) anode, Mg_0.07_V_2_O_5_·1.4H_2_O/rGO cathode, Whatman GF/D separator, and 0.5 M Mg(TFSI)_2_ in AN electrolyte in Ar glove box (H_2_O < 0.1 ppm, O_2_ < 0.1 ppm). Likewise, the full cell was assembled by the same cathode and separator in Ar glove box (H_2_O < 0.1 ppm, O_2_ < 0.1 ppm), while the 0.1-mm Mg foil and 0.4 M APC-CTAB were used as the anode and electrolyte. The electrochemical performance of both half-cells and full cells was evaluated at room temperature. Galvanostatic charge–discharge tests were carried out on a Neware-CT8000 with a voltage window of –1.2–1.4 V vs. AC for half-cell and 0.2–2.0 V vs. Mg^2+^/Mg for full cell. Cyclic voltammetry (CV) and electrochemical impedance spectroscopy (EIS) were evaluated on a CHI660E electrochemical workstation.

### Density Functional Theoretical Computations

Vienna ab initio simulation package (VASP) [36] was utilized to carry out density functional theoretical (DFT) calculations in the Perdew–Burke–Ernzerhof (PBE) formulation with projected augmented wave (PAW) potentials [37]. The 2 × 7 × 2 K-points and 500 eV of cutoff energy were used to fully optimize the structures. For vanadium, we combined the DFT + U methods to resolve the electron correlation effect, the U value was set to 3.25 eV. Regarding the structure, convergence was achieved when each atomic force was reduced to less than 0.01 eV Å^–1^. The Visualization for Electronic and Structural Analysis (VESTA) software was employed for the visualization and analysis of the crystal structure.

## Results and Discussion

### Structural Characterization of Mg_0.07_V_2_O_5_·1.4H_2_O/rGO

**Fig. 1:**
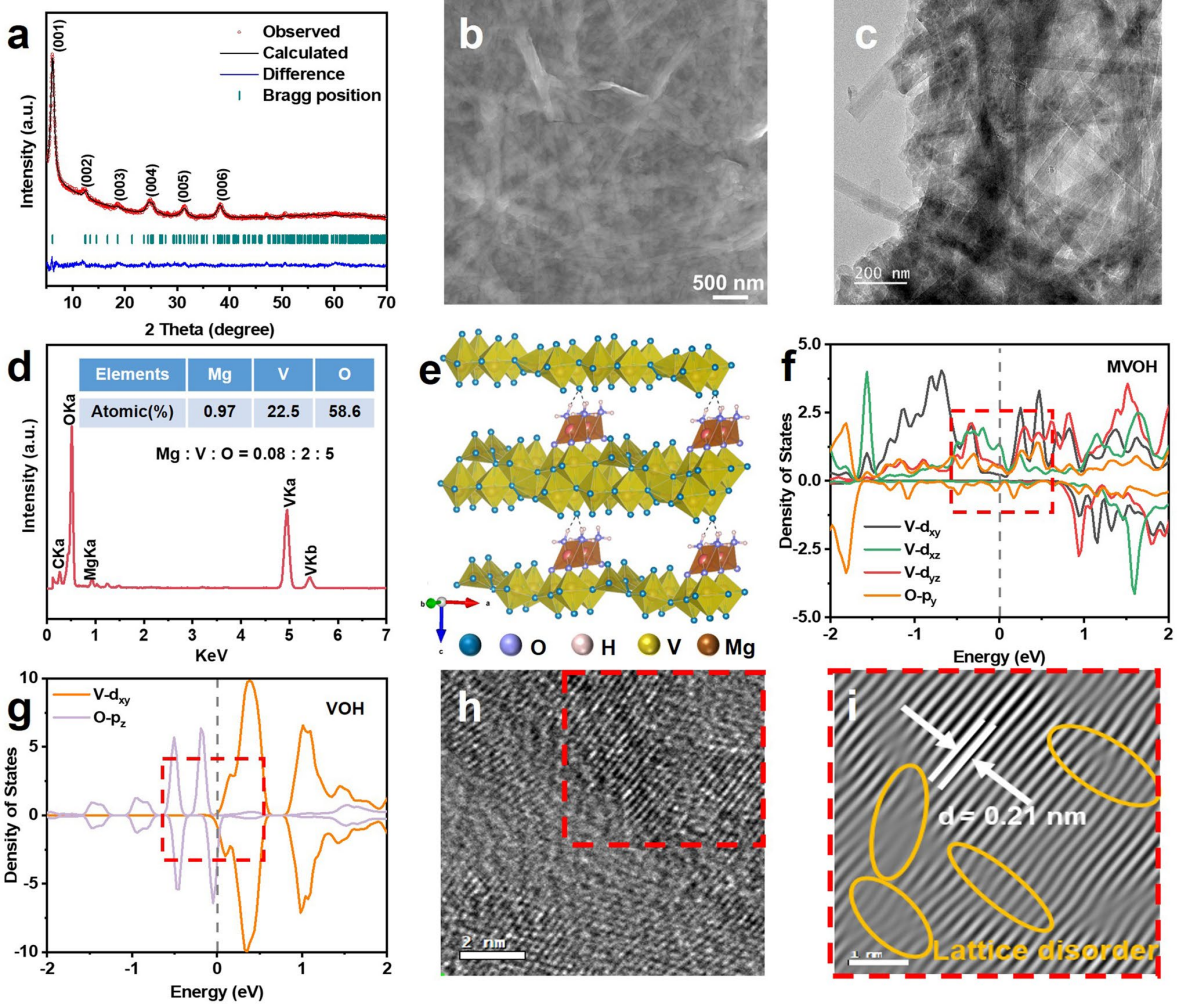
Characterization of MVOH/rGO. **a** Rietveld refinement of the XRD pattern. **b, c** SEM and TEM images. **d** EDS spectrum. **e** Lamellar crystal structure. **f** Partial density of states (PDOS) for V_2_O_5_·nH_2_O with P-Mg_d_. **g** PDOS for V_2_O_5_·nH_2_O without the defects. **h, i** HRTEM image and corresponding IFFT image

We employed a facile one-step hydrothermal method to synthesize rGO-substrated Mg_0.07_V_2_O_5_·1.4H_2_O nanobelts (MVOH/rGO) with dual defects, namely, interlayer P-Mg_d_ and surface O_d_ in V–O layers (Table S1). The XRD pattern shown in Fig. 1a displays a predominant diffraction peak at 6.1°, corresponding to the (001) plane, indicating a typical layered structure with an impressively large interlayer spacing of 14.5 Å [17, 38]. The Rietveld refinement method was employed to analyze the further detailed crystal structure of MVOH/rGO (Table S2), which reveals a monoclinic structure with space group C2/m, and the refined parameters are calculated to be *a* = 14.19 Å, *b* = 3.77 Å, and *c* = 14.34 Å; *α* = *γ* = 90°, and *β* = 83.9°. Notably, MVOH/rGO comprises anisotropic nanobelts several micrometers in length, and the finite width (50–70 nm) of the nanobelts effectively shortens the migration pathway of Mg^2+^ (Fig. 1b and c). The corresponding elemental mapping results (Figs. 1d and S1) confirm the presence of Mg, V, O, and C, aligning with the results of ICP–OES (Table S1). The P-Mg_d_ generates MgO_5_ pyramids between the V–O layers (Figs. 1e and S2). These MgO_5_ pyramids function as pins, fixing the lamellar structure of V_2_O_5_ in MVOH/rGO and thereby steadily restraining volume changes caused by Mg^2+^ insertion/extraction during discharging/charging. In addition, the P-Mg_d_ induces the polarization of V–O bonds [39]. According to ab initio calculations, this polarization leads to 3*d* orbital spitting, altering orbital hybridization in V–O bonds (Figs. 1f and S3a, b). Consequently, the valence band of V_2_O_5_ with P-Mg_d_ overlaps with its conduction band, resulting in substantially higher electronic conductivity of V_2_O_5_ in MVOH/rGO than that of V_2_O_5_·nH_2_O (Figs. 1g and S3c, d). Furthermore, the P-Mg_d_ causes the reduction of trace V^5+^ to V^4+^, as verified by the average vanadium valence of + 4.93 in MVOH/rGO according to the chemical formula of Mg_0.07_V_2_O_5_·1.4H_2_O (Fig. S5). The 3*d* electrons of V^4+^ can easily hop to the vacant 3*d* orbitals of V^5+^, further increasing the electronic conductivity of V_2_O_5_ nanobelts in MVOH/rGO. The O_d_ in the V–O layers is generated by rGO and reduces the negative charge density of the layers. This leads to looser bonding between the V–O layers and inserted Mg^2+^, resulting in a considerable interlayer spacing of 14.5 Å between the V–O layers of V_2_O_5_ in MVOH/rGO. In comparison, V_2_O_5_·1.5H_2_O (VOH) synthesized under similar conditions exhibits an interlayer spacing of 14.5 Å, while Mg_0.05_V_2_O_5_·1.0H_2_O (MVOH) synthesized under the same conditions with only P-Mg_d_ has an interlayer spacing as small as 13.5 Å (Fig. S6); this result shows the interlayer spacing enlargement effect of the O_d_ endowed by rGO [40]. The O_d_ also causes the reduction of superficial V^5+^ to V^4+^ at the interface between rGO and V_2_O_5_, further increasing the electronic conductivity of V_2_O_5_ in MVOH/rGO. According to the Raman analysis (Fig. S7), the signal intensity ratio of *sp*^2^ graphite carbon (D band) and *sp*^3^ disordered carbon (G band) in MVOH/rGO (*I*_D_/*I*_G_ = 1.14) is significantly higher than that in GO (*I*_D_/*I*_G_ = 0.8), implying that the deoxidization of GO is realized by the redox reaction between carboxyl groups in GO and V^5+^ [41, 44]. This is verified by the gas bubbles being observed after the hydrothermal synthesis of MVOH/rGO, which are attributed to the CO_2_ gas produced by the oxidation of carboxyl groups in GO. Due to the excellent electronic conductivity of rGO, the V_2_O_5_ nanobelts are connected by the conductive rGO network, ensuring rapid electrochemical insertion/extraction of Mg^2+^.

### Verification of Dual Defects in Mg_0.07_V_2_O_5_·1.4H_2_O/rGO

**Fig. 2:**
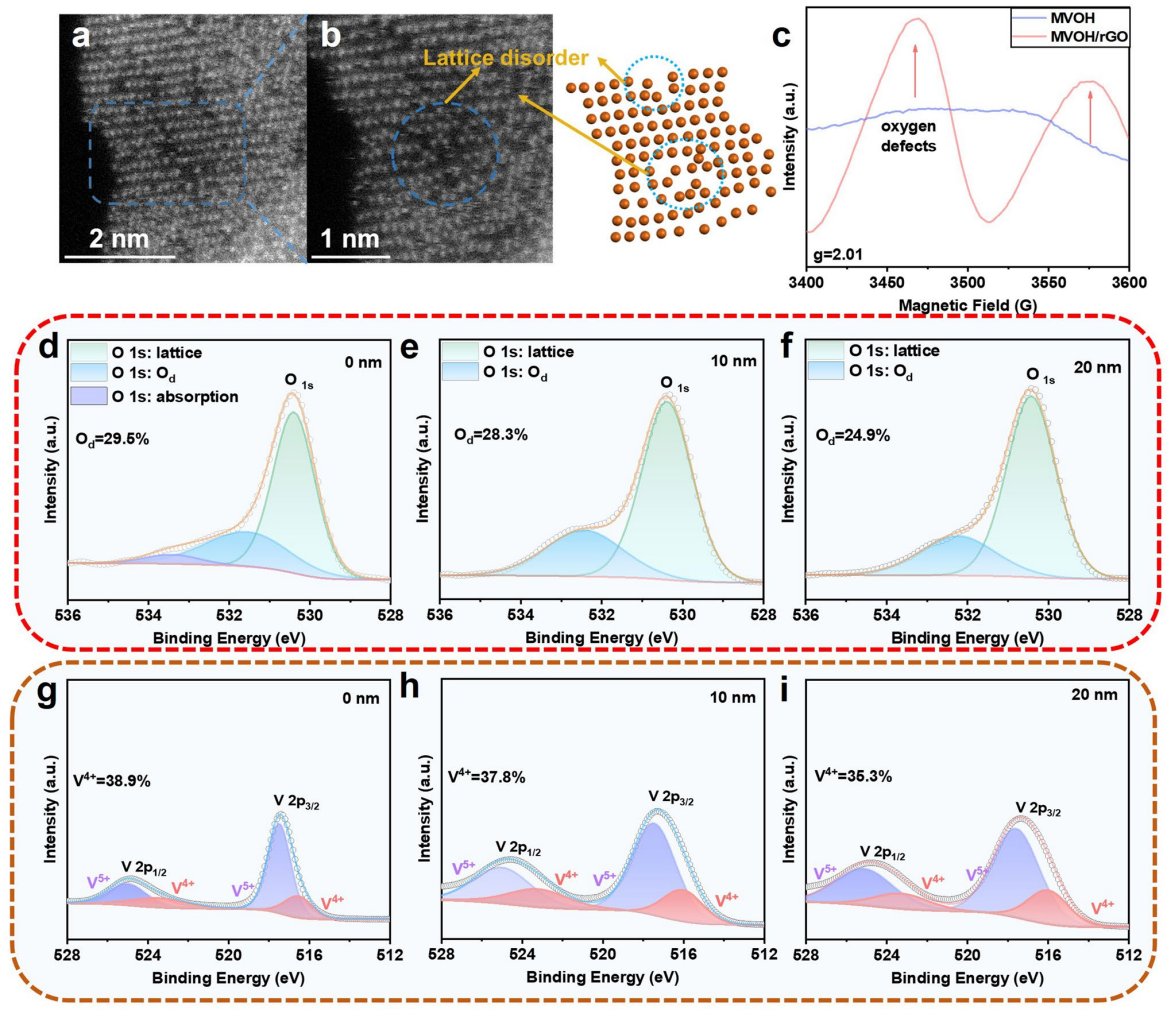
Verification of O_d_ in MVOH/rGO. **a, b** Atomic-resolution HAADF-STEM images and corresponding structural illustration. **c** EPR spectra of MVOH and MVOH/rGO. **d–f** High-resolution XPS spectra of O 1*s* at different etching depths. **g–i** High-resolution XPS spectra of V 2*p* at different etching depths

To confirm the presence of dual defects (P-Mg_d_ and O_d_) in MVOH/rGO, high-resolution transmission electron microscopy (HRTEM) was first employed (Fig. 1h and i). MVOH/rGO exhibited curved and discontinuous lattice fringes, indicating lattice disorder and suggesting the presence of lattice defects. Spherical aberration-corrected transmission electron microscopy (SAC-TEM) was employed to further investigate the lattice defects. As shown in Fig. 2a and b, V^5+^/V^4+^ vacancies are largely observed, implying the existence of O_d_. The results of the pair distribution function analysis shown in Fig. S8 reveal faint and indiscernible signals at > 24 Å, further confirming the existence of V^5+^/V^4+^ vacancies in MVOH/rGO. The O_d_ in MVOH/rGO was clearly detected at 3468 and 3576 G by electron paramagnetic resonance (EPR) characterization (Fig. 2c). In contrast, no O_d_ was detected in MVOH without rGO, confirming that rGO produces O_d_ in MVOH/rGO. Furthermore, argon ion etching X-ray photoelectron spectroscopy (A-XPS) was employed to investigate the dual defects in MVOH/rGO (Fig. S9). High-resolution XPS spectra of O 1*s* at an etching depth of 0 nm showed three characteristic peaks at 533.5, 531.6, and 530.4 eV, representing adsorbed oxygen, O_d_, and lattice oxygen, respectively [45, 47]. Results showed that the ratio of O_d_ decreases from 29.5% to 24.9% as the etching depth increases from 0 to 20 nm (Fig. 2d–f), demonstrating the presence of more O_d_ on the surface of V_2_O_5_ nanobelts than in the bulk, as O_d_ is produced by composited rGO. Similarly, moving from the MVOH/rGO surface to 20 nm inside, the V^4+^ content decreased from 38.9% to 35.3% (Fig. 2g–i), aligning with the decreasing trend of O_d_ content. The V^4+^ content in the superficial area of MVOH/rGO is substantially higher than the stoichiometric content of V^4+^ according to the Mg_0.07_V_2_O_5_ formula for MVOH/rGO and that in MVOH (Fig. S10), clearly confirming the coexistence of P-Mg_d_ and O_d_ in MVOH/rGO.

### Electrochemical Performance of Mg_0.07_V_2_O_5_·1.4H_2_O/rGO

**Fig. 3:**
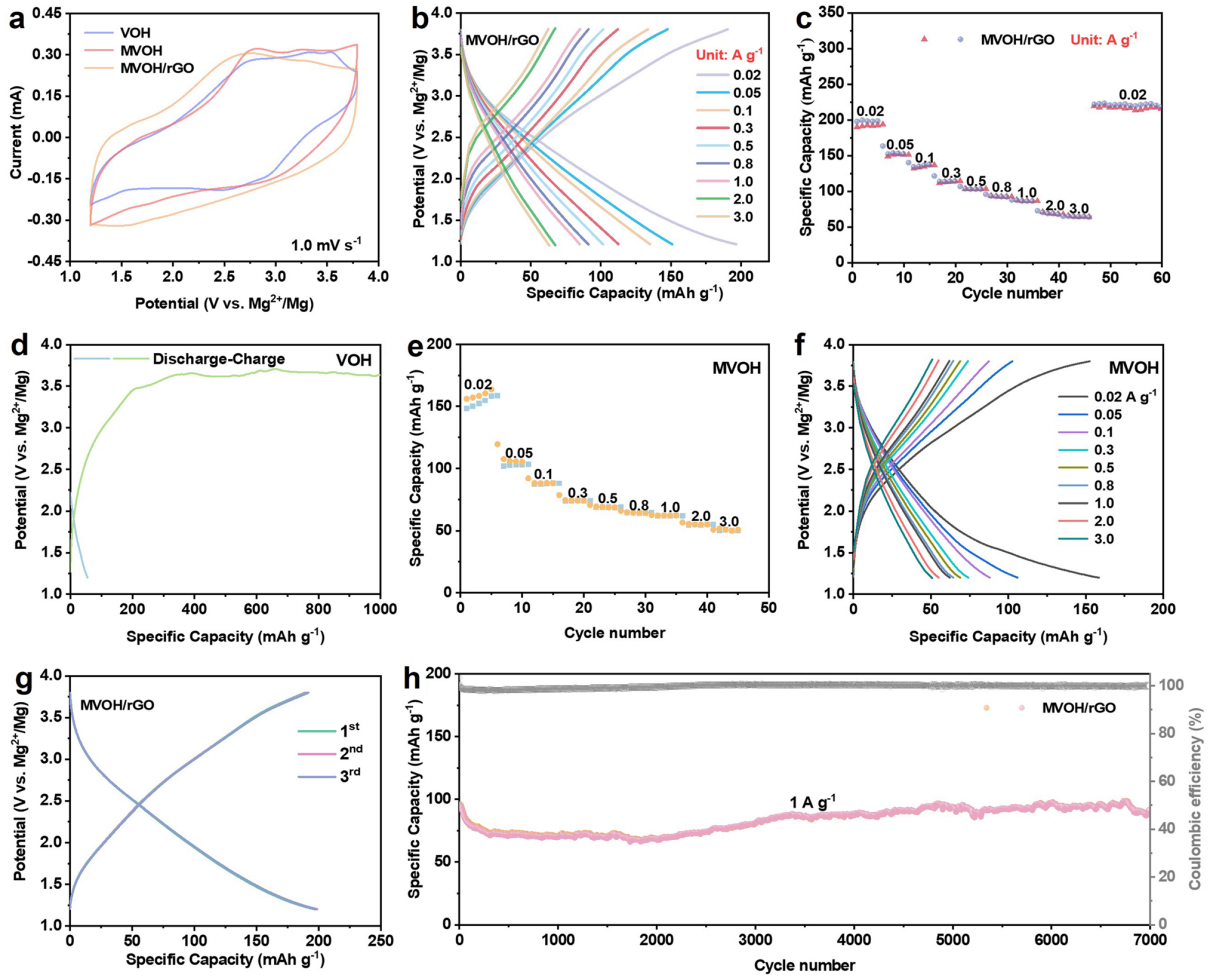
**a** CV curves of MVOH/rGO, MVOH, and VOH at 1.0 mV s^−1^. **b** GCD curves of MVOH/rGO at 0.02–3.0 A g^−1^. **c** Rate performance of MVOH/rGO at 0.02–3.0 A g^−1^. **d** GCD curves of VOH at 0.02 A g^−1^. **e, f** Rate performance and corresponding GCD curves of MVOH. **g** GCD curves of MVOH/rGO in the initial three cycles at 0.02 A g^−1^. **h** Long-term cycle performance of MVOH/rGO at 1 A g^-1^

The Mg^2+^ storage performance of MVOH/rGO as the cathode material with dual defects was investigated. A coin cell was assembled using activated carbon as the anode and 0.5 M Mg(TFSI)_2_ in acetonitrile as the electrolyte, with the activated carbon anode having a stable potential and simultaneously functioning as a reference electrode and counter electrode [48]. The cyclic voltammetry (CV) curve of the MVOH/rGO cathode exhibited the lowest and highest redox peaks at 1.5/2.7 V and maximum area within the potential window of 1.2–3.8 V (vs. Mg^2+^/Mg) at 1.0 mV s^−1^ (Fig. 3a). Moreover, the peak potentials of these redox peaks are in high accordance with the galvanostatic charge/discharge (GCD) curves shown in Fig. 3b, indicating superior charge-transfer efficiency and Mg^2+^ storage capacity of the MVOH/rGO cathode compared to VOH and MVOH. Consequently, the MVOH/rGO cathode exhibits a superior discharge capacity of 197 mAh g^−1^ at 0.02 A g^−1^ (Fig. 3c), corresponding to 0.15 mol of Mg^2+^ inserted per mole of V_2_O_5_ [5]. The chemical reaction for magnesiation is as follows:

1 $${\text{Mg}}_{{{0}{\text{.07}}}} {\text{V}}_{{2}} {\text{O}}_{{5}} \cdot {1}{\text{.4H}}_{{2}} {\text{O + 0}}{\text{.15Mg}}^{{2 + }} { + 0}{\text{.3e}}^{ - } { } \leftrightarrow {\text{ Mg}}_{{{0}{\text{.22}}}} {\text{V}}_{{2}} {\text{O}}_{{5}} \cdot {1}{\text{.4H}}_{{2}} {\text{O}}$$

As the current density increases to 3.0 A g^−1^, the MVOH/rGO cathode still exhibits a considerable rate performance of 64 mAh g^−1^. In contrast, VOH without defects cannot discharge and charge with acceptable Coulombic efficiency (CE), which is due to the decomposition of electrolyte; according to the charging curve in Fig. 3d, the VOH cathode cannot be charged to higher than 3.5 V, and the electrolyte decomposed when the voltage is higher than 3.5 V. However, according to the initial GCD curves for MVOH and MVOH/rGO (Fig. S11), the decomposition of electrolyte was not observed for MVOH and MVOH/rGO cathodes. MVOH with only P-Mg_d_ shows increased electronic conductivity due to the overlap of its conduction and valence bands. However, its interlayer spacing is smaller than that of MVOH/rGO, resulting in an inferior rate capability (148/51 mAh g^−1^ at 0.02/3.0 A g^−1^; Fig. 3e) compared to that of MVOH/rGO. The GCD curves of MVOH display standard surface adsorption behavior without a redox plateau, indicating a capacity lower than that of MVOH/rGO (Fig. 3f). Owing to the pinning effect of P-Mg_d_, the MVOH/rGO cathode presents remarkably similar GCD curves in the initial three cycles at 0.02 A g^−1^, consistent with the CV results shown in Fig. S12, indicating the high reversibility of Mg^2+^ insertion/extraction (Fig. 3g). In addition, the MVOH/rGO cathode exhibits a notable capacity retention of 103 mAh g^−1^ after 900 cycles at 0.5 A g^−1^ with a capacity decay rate of only 0.014% per cycle, demonstrating its high cycle stability (Fig. S14a). In comparison, the VOH-O_d_ cathode (Fig. S13) exhibits a significantly lower cycle capacity of 57 mAh g^−1^ at 0.5 A g^−1^ and experiences much higher capacity decay rate of 0.04% per cycle (Fig. S14b); moreover, the MVOH cathode exhibits a substantial capacity decline (0.05% per cycle), remaining at a low capacity of 66 mAh g^−1^ after 900 cycles at 0.5 A g^−1^ (Fig. S14c). This comparison highlights the disability of P-Mg_d_ or O_d_ alone to improve the cathode cycle lifespan. Furthermore, even at a higher current density of 1.0 A g^−1^, the MVOH/rGO cathode can still maintain an impressive capacity of 91 mAh g^−1^ with an average CE of 99.6% after cycling for over 500 h (Figs. 3h and S15). The durability of the dual-defect MVOH/rGO cathode is higher than those of previously reported cathodes (Table S3), including PEDOT-intercalated V_2_O_5_ [10], O_d_-V_2_O_5-x_ [49], and NaV_2_O_2_(PO_4_)_2_F composited with rGO [50]. These results demonstrate the success of dual-defect engineering in prolonging the cathode cycle lifespan.

**Fig. 4:**
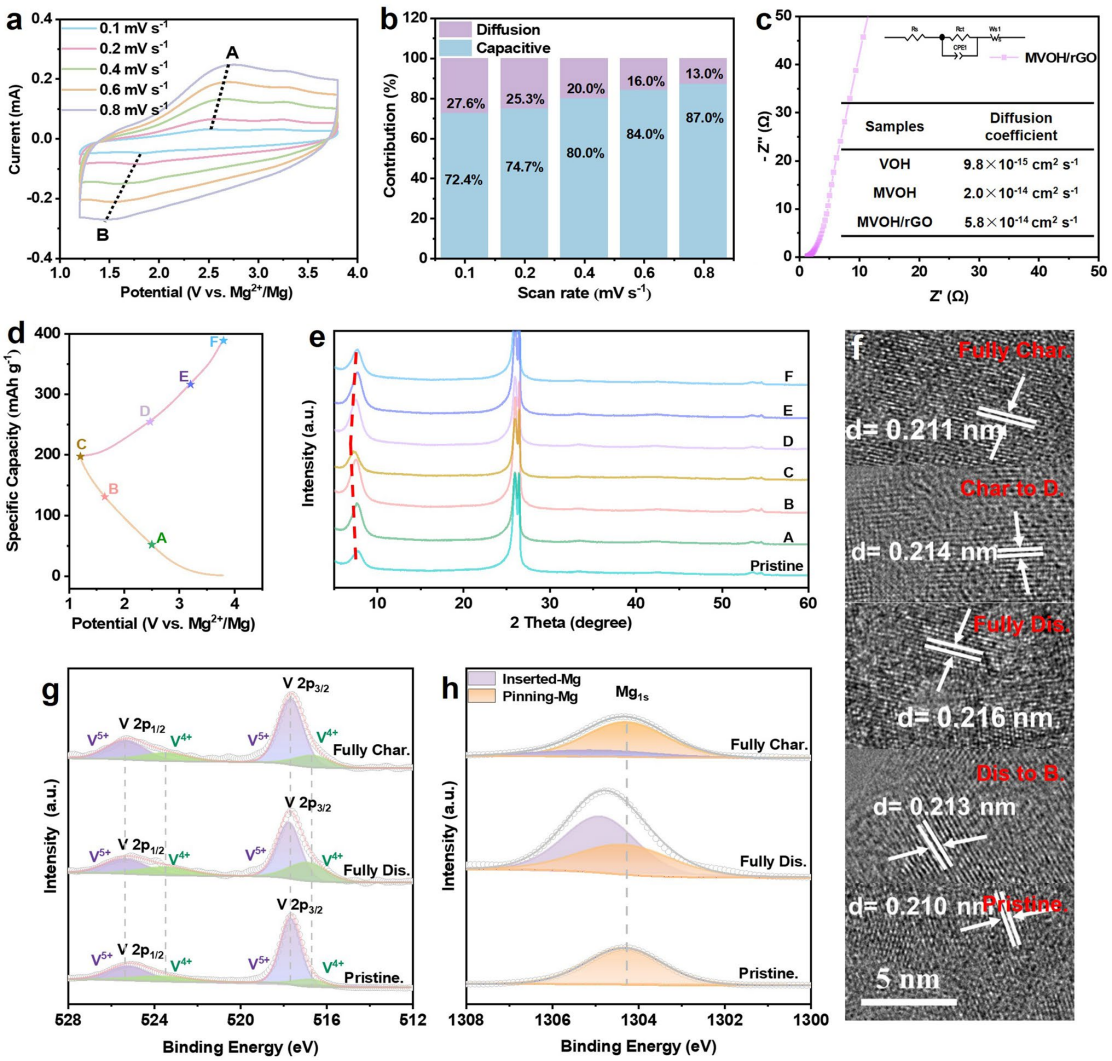
Charge-storage mechanism of the MVOH/rGO cathode. **a** CV curves at scan rates of 0.1–0.8 mV s^−1^. **b** Capacitive and diffusion contribution ratio at 0.1–0.8 mV s^−1^. **c** EIS spectrum. **d** GCD profiles at 0.02 A g^−1^. **e** Ex situ XRD spectra at different charge/discharge states (A–F). **f** HRTEM images obtained in different charge–discharge states. **g, h** High-resolution ex situ XPS spectra of V 2*p* and Mg 1*s* in fully charged, fully discharged, and pristine states

To investigate the Mg^2+^ storage mechanism of the dual-defect MVOH/rGO cathode, CV tests were conducted at scan rates of 0.1–0.8 mV s^−1^ (Fig. 4a). The redox peaks exhibited a constant shape as the response currents expanding. The voltammetric response of the MVOH/rGO cathode follows the equation *i* = *av*^*b*^, where “*i*” represents the peak current, and *a* and *b* are adjustable constants [51, 52]. Further, *b* = 1 indicates a capacitive-controlled process, while *b* = 0.5 indicates a diffusion-controlled process. For MVOH/rGO, the *b* values of peaks A and B at various scan rates are 0.96 and 0.81, respectively, indicating that the Mg^2+^ storage process is dominated by capacitive behavior (Fig. S16). Compared with VOH without defects and MVOH with only P-Mg_d_, the contribution ratio of pseudocapacitive reactions in MVOH/rGO increases, which demonstrates the positive effect of dual defects in enhancing the capacitive contribution (Fig. S17). At various scan rates, the capacitive contribution in MVOH/rGO is > 72% (Fig. 4b), consistently surpassing those in VOH and MVOH. This underscores the superior ionic/electronic conductivity elicited by the dual defects. Owing to the dual defects, ion and electron diffusion in the MVOH/rGO cathode are rapid, and the charge-storage process is solely controlled by surface pseudocapacitive reactions, indicating fast Mg^2+^ insertion/extraction and the compatibility of the cathode with magnesium-metal anodes. The charge transport dynamics in MVOH/rGO were analyzed by electrochemical impedance spectrometry (EIS) measurements. As anticipated, the MVOH/rGO cathode exhibited the smallest charge-transfer resistance (*R*_ct_), confirming its highest ionic/electronic conductivity (Figs. 4c and S18). The Mg^2+^ diffusion coefficient (${\text{D}}_{{\text{Mg}}^{2+}}$) of MVOH/rGO was as high as 5.8 × 10^−14^ cm^2^ s^−1^, substantially surpassing those of VOH (9.8 × 10^−15^ cm^2^ s^−1^) and MVOH (2.0 × 10^−14^ cm^2^ s^−1^), indicating the highest ionic conductivity of MVOH/rGO.

To further investigate the charge-storage mechanism, we conducted ex situ XRD, XPS, and TEM characterizations. Figure 4d and e shows the XRD patterns of the MVOH/rGO cathode at different discharge/charge states (A–F). No new phase was detected; however, the (001) diffraction peak slightly shifted during discharging/charging. In particular, the (001) diffraction peak shifted toward a lower angle for States A–C, indicating increased spacing between the V–O layers. During charging in States C–F, the (001) diffraction peak shifted back to its original location, indicating full recovery of the interlayer spacing after Mg^2+^ extraction. This demonstrates the high reversibility and cycle stability of the MVOH/rGO cathode with dual defects. In addition, the variation in the (001) peak location is only within 1°, verifying the high structural stability provided by the pinning effect of P-Mg_d_. Figure 4f shows a slight increase in lattice spacing by 0.03 Å (from 0.210 to 0.213 nm) at the discharged State B, and the spacing further expands to 0.216 nm at fully discharged state with total variation ratio less than 3%. Subsequently, upon charging to charged State D, the interlayer spacing contracts to 0.214 nm and further decreases to 0.211 nm at full dicharged state. Such a negligible interlayer spacing variation further confirms that the combination of the pinning effect of P-Mg_d_ and the interlayer force relaxation effect of O_d_ can effectively restrain irreversible structural changes (Figs. S19 and S20). Figure 4g and h shows the high-resolution XPS spectra of V 2*p* and Mg 1*s*. The valence percentages of V^4+^ and V^5+^ in the pristine MVOH/rGO cathode are 27% and 73%, respectively. In the fully discharged state, the V^4+^ content increased to 56%, aligning with the reduction process accompanied by Mg^2+^ insertion. After full charge, the V^4+^ content decreased to 32%, which corresponds to the oxidation reaction during Mg^2+^ extraction. Compared to the pristine MVOH/rGO cathode, the slight increase in the V^4+^ content in fully charged MVOH/rGO indicates the presence of trace inserted Mg^2+^. The V^4+^ ratio in Fig. 4g is lower than that in Fig. 2g is due to the oxidation of partial surface V^4+^ during the electrode preparation process, which does not influence the relative variation trend of V^4+^ ration during charing/discharging. The Mg 1*s* signal at 1304.3 eV corresponded to the P-Mg_d_ species in MVOH/rGO, indicating that all pre-intercalated Mg^2+^ was pinned to the lamellar structure of MVOH/rGO (Fig. 4h). In the fully discharged state, a new signal appeared at 1304.9 eV, which was attributed to electrochemically inserted Mg^2+^. The presence of these two different chemical environments of Mg^2+^ is due to the distinct interaction modes of Mg^2+^ with the lattice O atoms [5]. After full charge, the signal intensity of inserted Mg^2+^ substantially decreased, with trace inserted Mg^2+^ remaining in the interlayer, which is in agreement with previously reported results [53, 54].

**Fig. 5:**
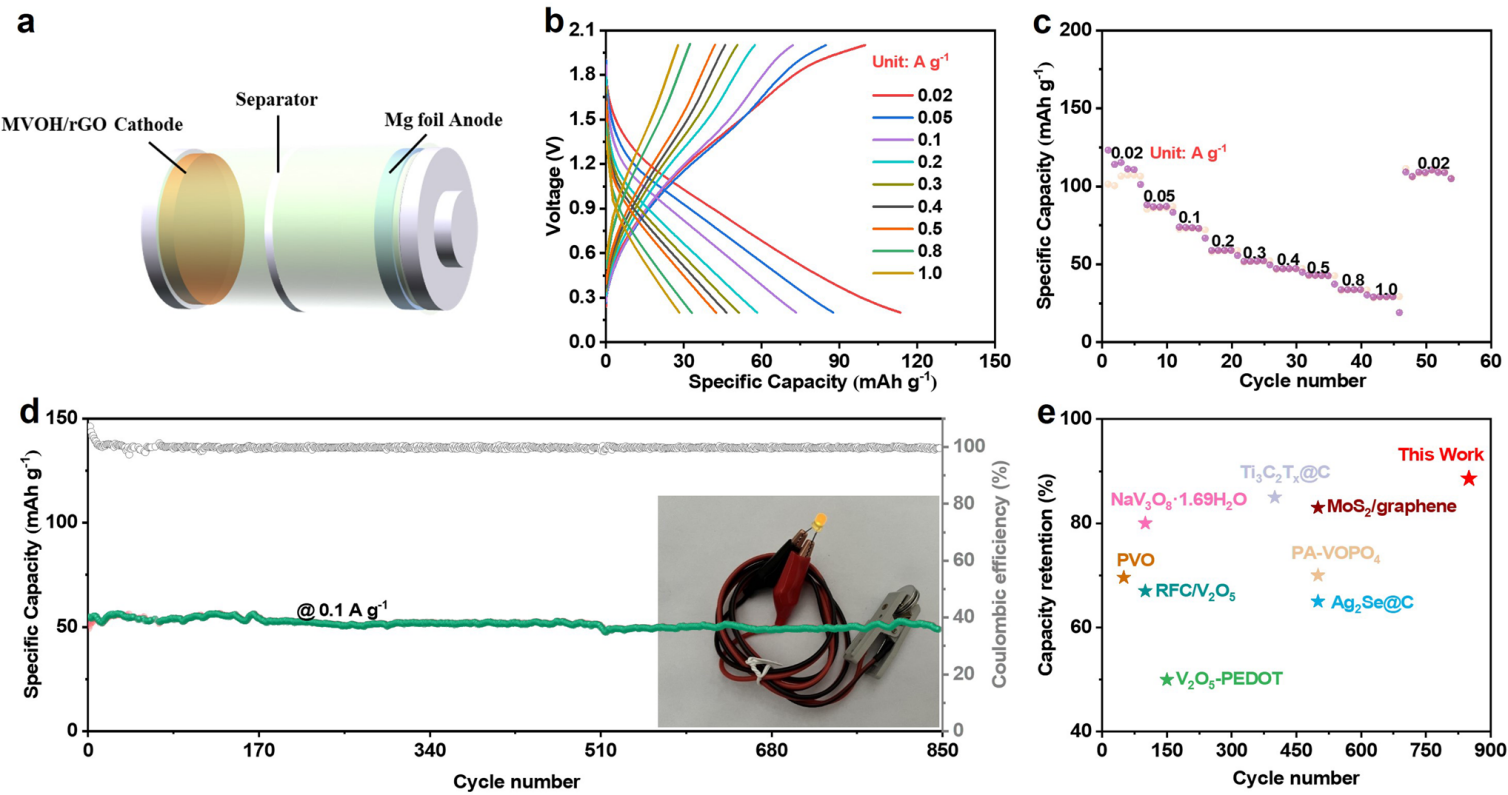
Electrochemical performance of the Mg foil//MVOH/rGO full cell. **a** Schematic of the cell. **b** GCD curves at a current density of 0.02–1.0 A g^−1^. **c** Rate performance. **d** Long-term cycle performance at 0.1 A g^−1^, Inset: powering an orange LED. **e** Comparison results of the cycle lifespan

To comprehensively evaluate the performance of the MVOH/rGO cathode, we assembled a Mg foil//MVOH/rGO full cell using 0.4 M APC-CTAB electrolyte because the magnesium-metal anode tends to passivate in the Mg(TFSI)_2_ electrolyte (Fig. 5a) [55, 56]. Leveraging the favorable ionic and electronic diffusion performance of MVOH/rGO, the Mg foil//MVOH/rGO full cell demonstrated a considerable initial discharge capacity of 123 mAh g^−1^ at 0.02 A g^−1^, operating at 1.1 V and exhibiting an energy density of 86 Wh kg^−1^ (Fig. 5b). Even with an increased current density to 1.0 A g^−1^, it retained a capacity of 29 mAh g^−1^, showcasing superior rate performance (Fig. 5c). The dual-defect engineering of MVOH/rGO significantly restricted volume changes during magnesiation/demagnesiation, leading to an outstanding capacity retention of 75 mAh g^−1^ after 400 cycles at 0.05 A g^−1^, with a slight capacity decay of 0.027% per cycle and an average CE as high as 99.5% (Fig. S21). At a current density of 0.1 A g^−1^, the cell maintained an impressive lifespan of 850 cycles, with a capacity retention ratio of 84% (Fig. 5d). Furthermore, it could continuously power an orange LED, showcasing its promising practical applications. In comparison with those of previously reported vanadium-based or carbon-substrated cathode materials, such as RFC/V_2_O_5_ [57], V_2_O_5_-PEDOT [23], PA-VOPO_4_ [9], PVO [22], NaV_3_O_8_·1.69H_2_O [58], MoS_2_/graphene [59], Ag_2_Se@C [60], and Ti_3_C_2_T_x_@C [61], the cycle lifespan of the dual-defect MVOH/rGO cathode is significantly higher (Fig. 5e and Table S4).

## Conclusions

In conclusion, we propose dual-defect engineering to enhance the cycle lifespan and rate capability of lamellar RMMB cathodes. Utilizing lamellar V_2_O_5_·nH_2_O as a demo cathode material, we synthesized an MVOH/rGO cathode featuring dual defects, namely, O_d_ and P-Mg_d_. The O_d_ serves to weaken the interactions between Mg^2+^ and the V–O layers, increasing the interlayer spacing. This augmentation accelerates the Mg^2+^ migration kinetics and prevents structural collapse. Further, the P-Mg_d_ secures the lamellar structure with a large interlayer spacing, promoting favorable Mg^2+^ migration kinetics and realizing high structural stability. Inducing the overlap of the conduction and valence bands enhances electronic conductivity and elevates the cathode’s performance in RMMBs. Consequently, the developed Mg foil//MVOH/rGO full cell demonstrates an exceptional lifespan of 850 cycles at 0.1 A g^−1^, with a capacity retention ratio of 84%. This innovative approach opens a new avenue for developing high-durability, high-capacity cathodes, thereby advancing the practical application of RMMBs and other new secondary batteries. The proposed dual-defect engineering strategy can be generalized to other cathode materials to enhance the performance of RMMB batteries.

## Supplementary Information

Below is the link to the electronic supplementary material.

Supplementary file1 (DOCX 4110 KB)
