# Supplementary material for: Dual-Defect Engineering Strategy Enables High-Durability Rechargeable Magnesium-Metal Batteries
Source: Nanomicro Lett. 2024 Apr 29;16:184. doi: 10.1007/s40820-024-01410-8 (PMC11058737; doi:10.1007/s40820-024-01410-8)
Supplement: Supplementary file 1 — Supplementary file1 (DOCX 4110 KB) [file 40820_2024_1410_MOESM1_ESM.docx]

Supporting Information for

**Dual-Defect Engineering Strategy Enables High-Durability Rechargeable Magnesium-Metal Batteries**

Fuyu Chen^1,2^, Bai-Qing Zhao^3^, Kaifeng Huang^1,2^, Xiu-Fen Ma^1,2^, Hong-Yi Li^1,2,^*, Xie Zhang^4^, Jiang Diao^1,2^, Jili Yue^1,2^, Guangsheng Huang^1,2^, Jingfeng Wang^1,2^, Fusheng Pan^1,2,5,^*

^1^ National Innovation Center for lndustry-Education Integration of Energy Storage Technology, School of Materials Science and Engineering, Chongqing University, Chongqing 400044, P. R. China

^2^ National Magnesium Alloy Material Engineering Technology Research Center, Chongqing University, Chongqing 400044, P. R. China

^3^ Materials and Energy Division, Beijing Computational Science Research Center, Beijing 100193, P. R. China

^4^ School of Materials Science and Engineering, Northwestern Polytechnical University, Xi'an 710072, P. R. China.

^5^ National Key Laboratory of Advanced Casting Technologies, Chongqing University, Chongqing 400044, P. R. China

*Corresponding authors. E-mail: [hongyi.li@cqu.edu.cn](mailto:hongyi.li@cqu.edu.cn) (Hong-Yi Li); [fspan@cqu.edu.cn](mailto:fspan@cqu.edu.cn) (Fusheng Pan)

**Supplementary Figures and Tables**


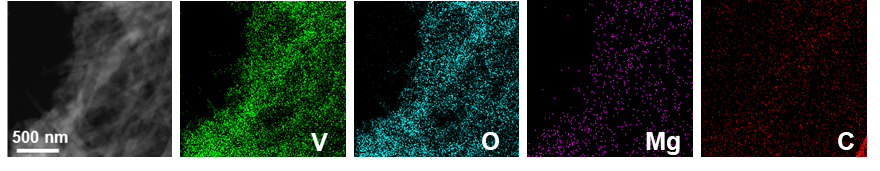


**Fig. S1** Elemental mapping results of V, O, Mg, and C in MVOH/rGO


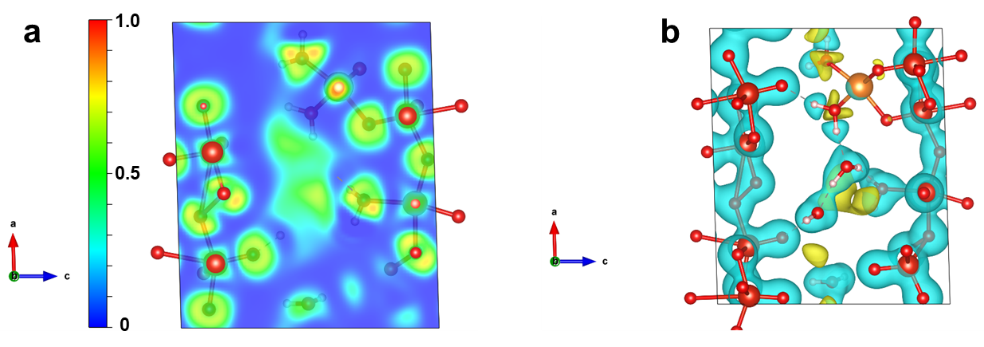


**Fig. S2** The Electron Localization Function simulations results of (**a**) and Charge Density Difference of (**b**)

Electron Localization Function (ELF) results in **Fig. S2a** demonstrate strong localization of electrons around Mg, suggesting an increased tendency for these electrons to transfer from O, and the large ELF on Mg−O bond confirms the presence of strong interactions between Mg and O. Subsequently, the calculated Charge Density Difference (CDD) in **Fig. S2b** reveals charge accumulation around Mg (represented by the blue electron cloud) and corresponding electron loss around O (depicted by the yellow electron cloud), this suggests that O in V−O layers can donate its lone pair electrons to the vacant orbitals of P-Mg_d_, indicating a strong tendency to generate coordination bonds between Mg and O from V−O layers. Therefore, it is demonstrated that the bonding occurs between P-Mg_d_ and O in the V−O layers, leading to the generation of MgO_5_ polyhedra.


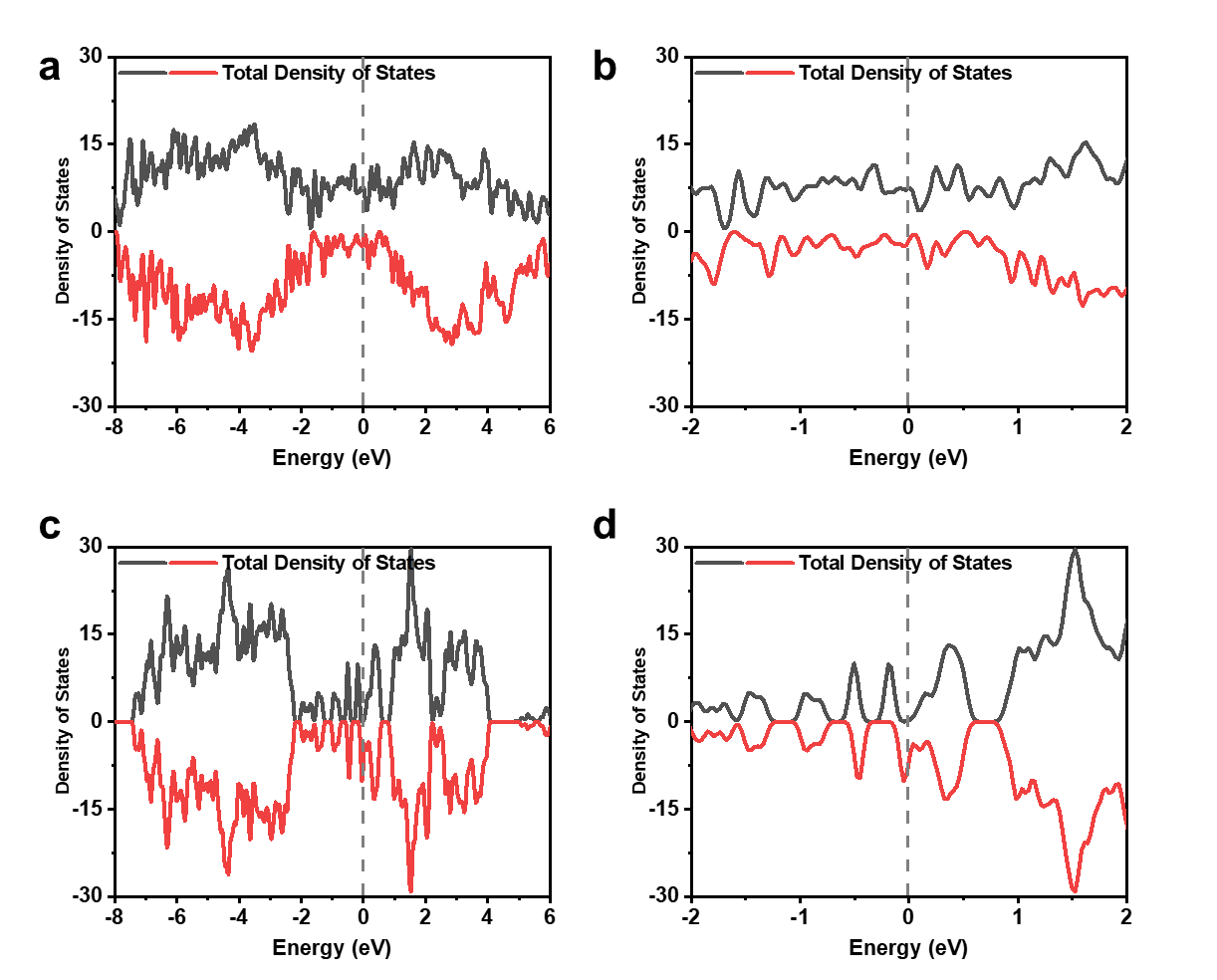


**Fig. S3** Total Density of States (TDOS) of (**a, b**) MVOH and (**c, d**) VOH

The TDOS of MVOH (**Fig. S3a** and **S3b)** demonstrates a higher density of electronic states near the Fermi level compared to VOH (**Fig. S3c** and **S3d)**, verifying the enhancement of conductivity attributed to the P-Mg_d_ in V_2_O_5_·nH_2_O.


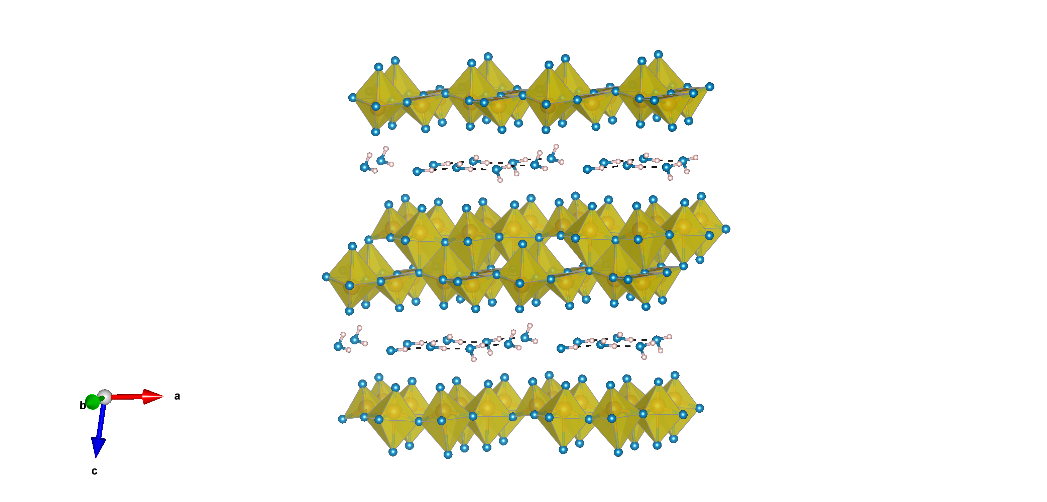


**Fig. S4** Lamellar crystal structure of VOH


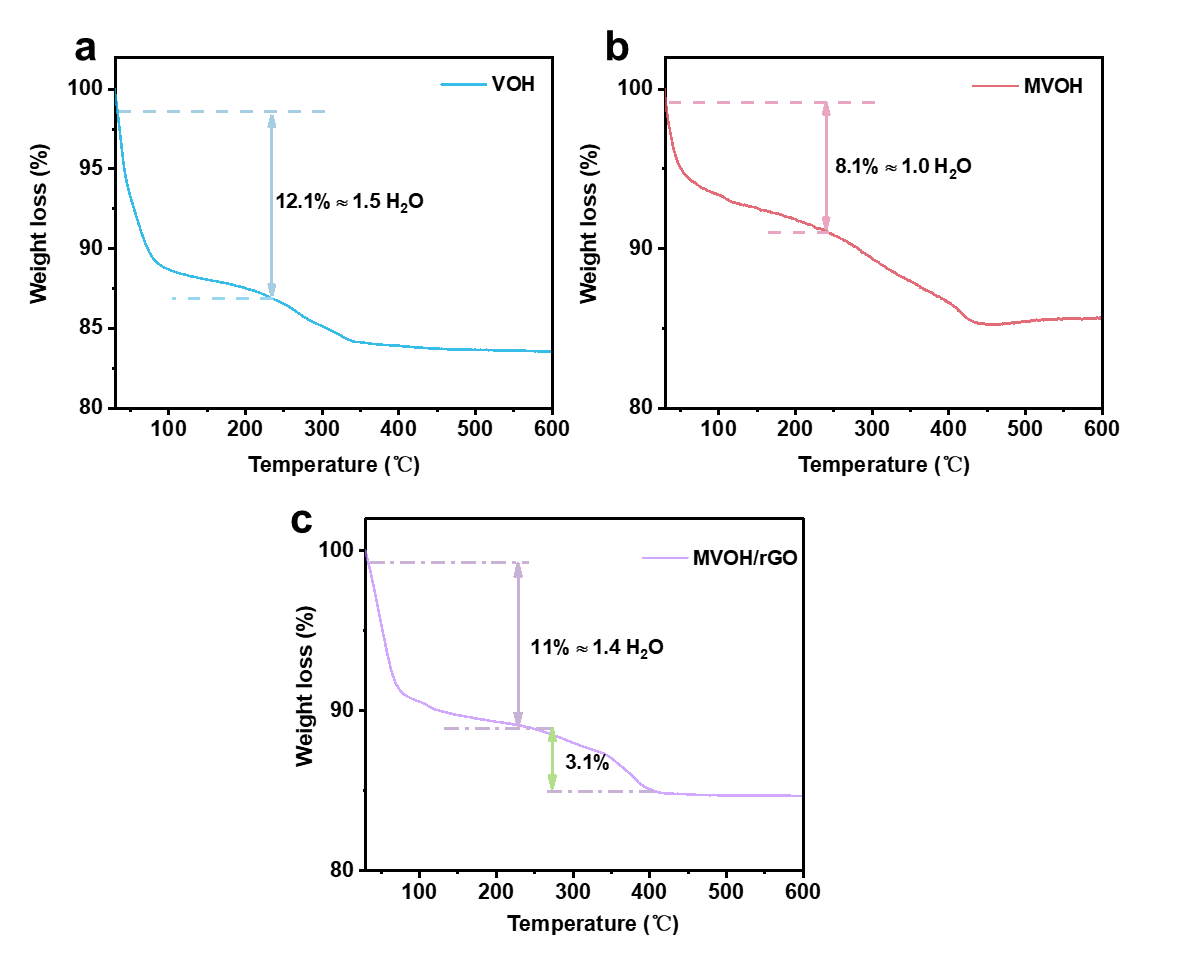


**Fig. S5** TG profiles of (**a**) VOH, (**b**) MVOH, and (**c**) MVOH/rGO

Results show that pristine VOH contains 1.5 crystal water molecules per unit. The crystal water content of MVOH decreases to 1.0 because the interaction force between the pre-interalated Mg^2+^ and V–O layers shrinks the interlayer spacing. However, O_d_ can greatly alleviate the interaction caused by P-Mg_d_, and the crystal water content of MVOH/rGO thus recovers to 1.4 per unit. Furthermore, the 3.1% weight loss observed in **Fig. S5c** between 270°C and 400°C can primarily be attributed to the thermal degradation of rGO in MVOH/rGO composite. The rGO content is thus deduced to be 11.4% in MVOH/rGO composite, with Mg_0.07_V_2_O_5_·1.4H_2_O content of 88.6%.

**Fig. S6** XRD patterns of VOH, MVOH, and MVOH/rGO


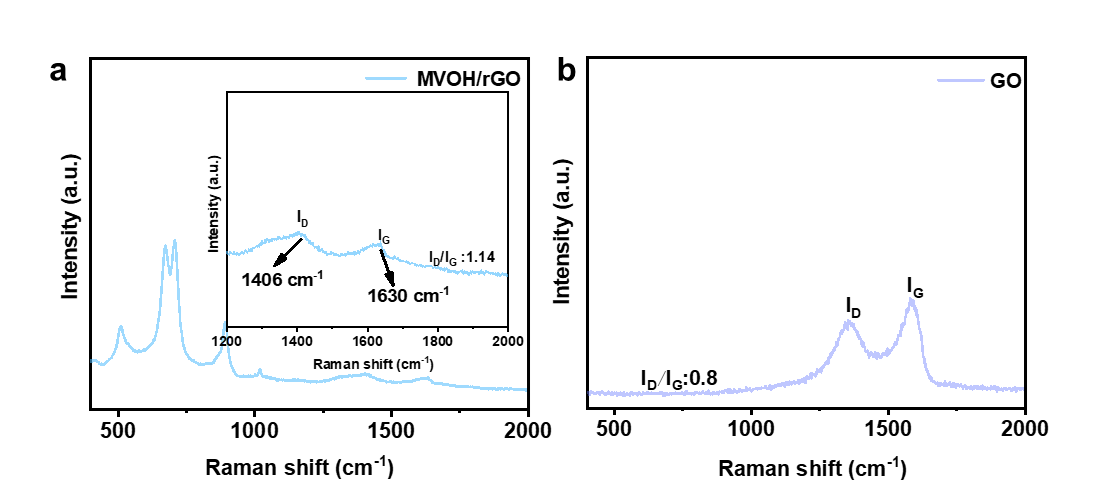


**Fig. S7** Raman spectra of (**a**) MVOH/rGO and (**b**) GO


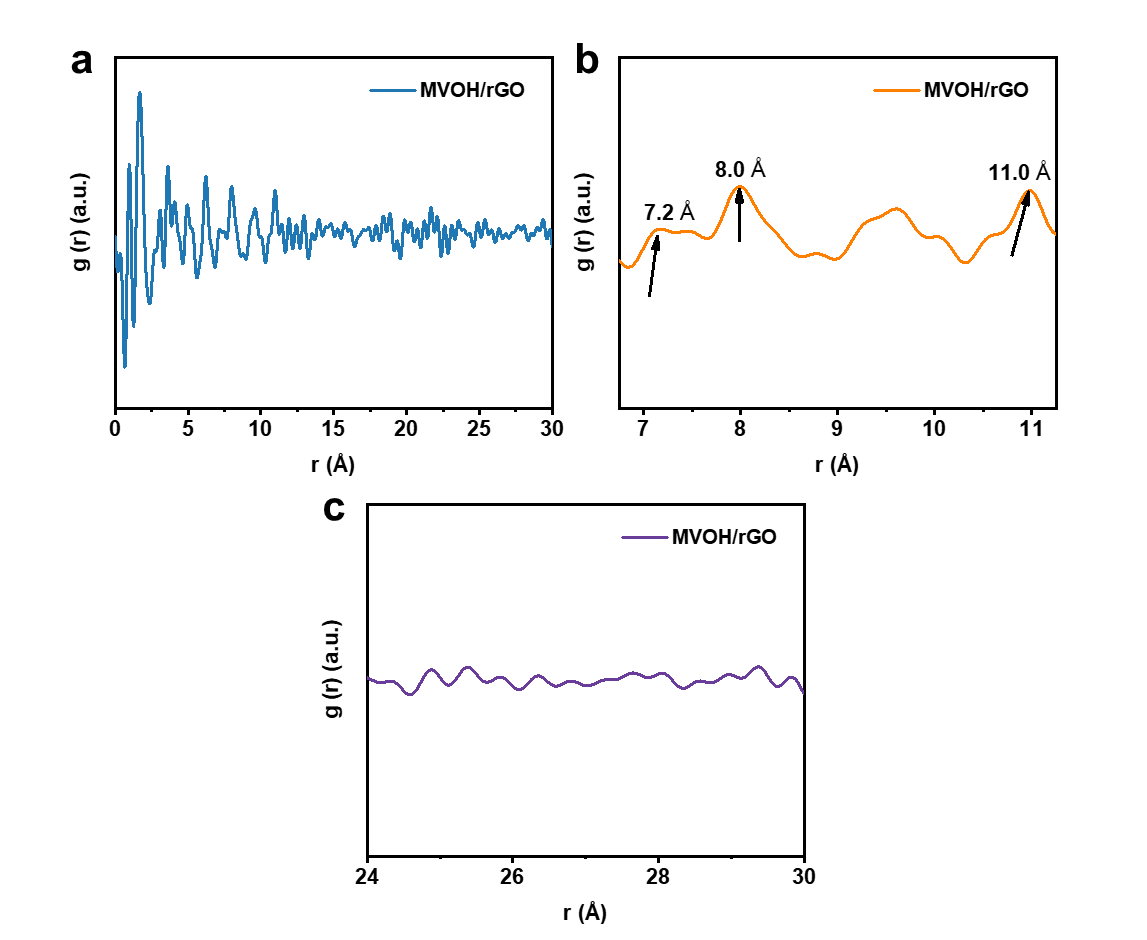


**Fig. S8** (**a−c**) The pair distribution function results of MVOH/rGO at different midden range

The PDF analysis results at 5-10 Å in **Fig. S8a** reveal the typical characterization of V_2_O_5_ [S1], in which the peaks at 7.2 Å, 8.0 Å, and 11.0 Å in **Fig. S8b** represent the interatomic distance of V**−**V along the b direction and the a direction, respectively.

**Fig. S9** XPS survey spectra of MVOH/rGO at the Ar ion etching depth of 0, 10, 20 nm

In agreement with the ICP–OES and EDX analysis results, the XPS survey spectra in **Fig. S9** shows the signals of V, Mg, O, and C. The signal intensity of C element decreases as the etching depth increasing from 0 to 20 nm, while the signals of V, Mg, and O elements increase, which verifies the surface contact between MVOH and rGO.


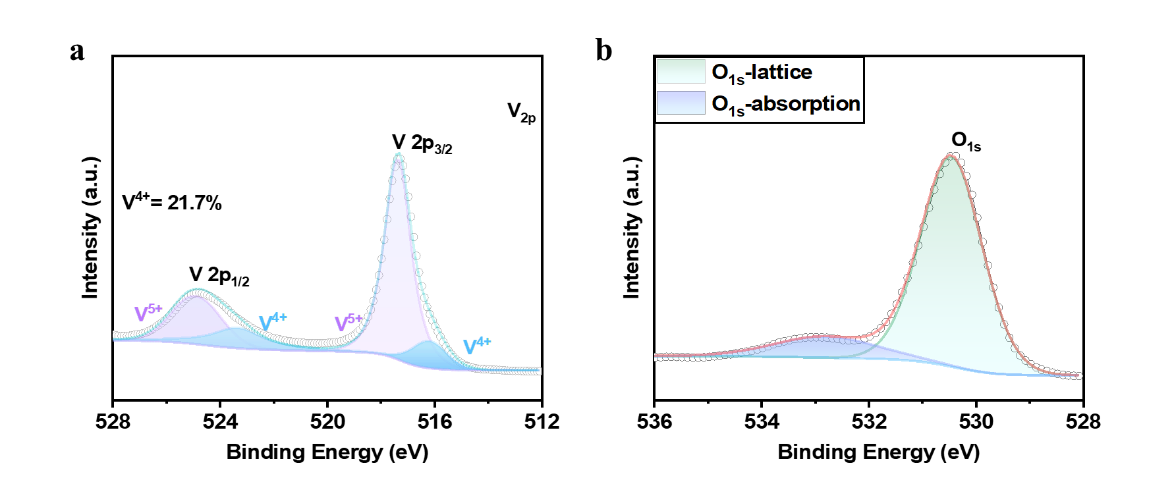


**Fig. S10** (**a**) V 2p and (**b**) O 1s high-resolution XPS spectra of MVOH


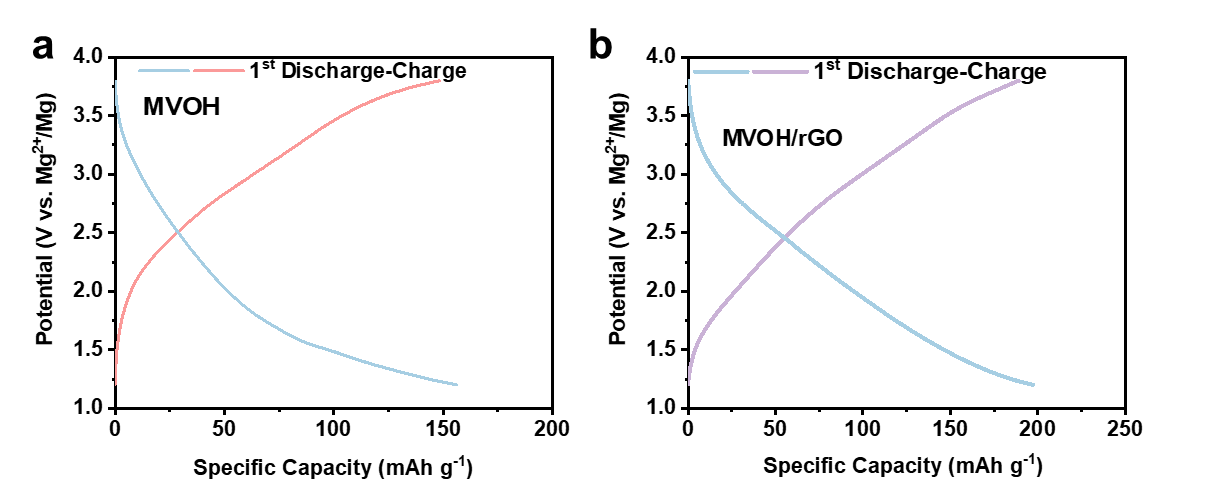


**Fig. S11** Initial GCD curves at 0.02 A g^−1^ of (**a**) MVOH and (**b**) MVOH/rGO cathodes

**Fig. S12** CV curves of MVOH/rGO at 0.1 mV s^−1^ for the initial three cycles

**Fig. S13** EPR spectrum of VOH-O_d_

As shown in **Fig. S13**, the characteristic peaks of O_d_ were clearly observed, implying the existence of O_d_ in VOH-O_d_.

**
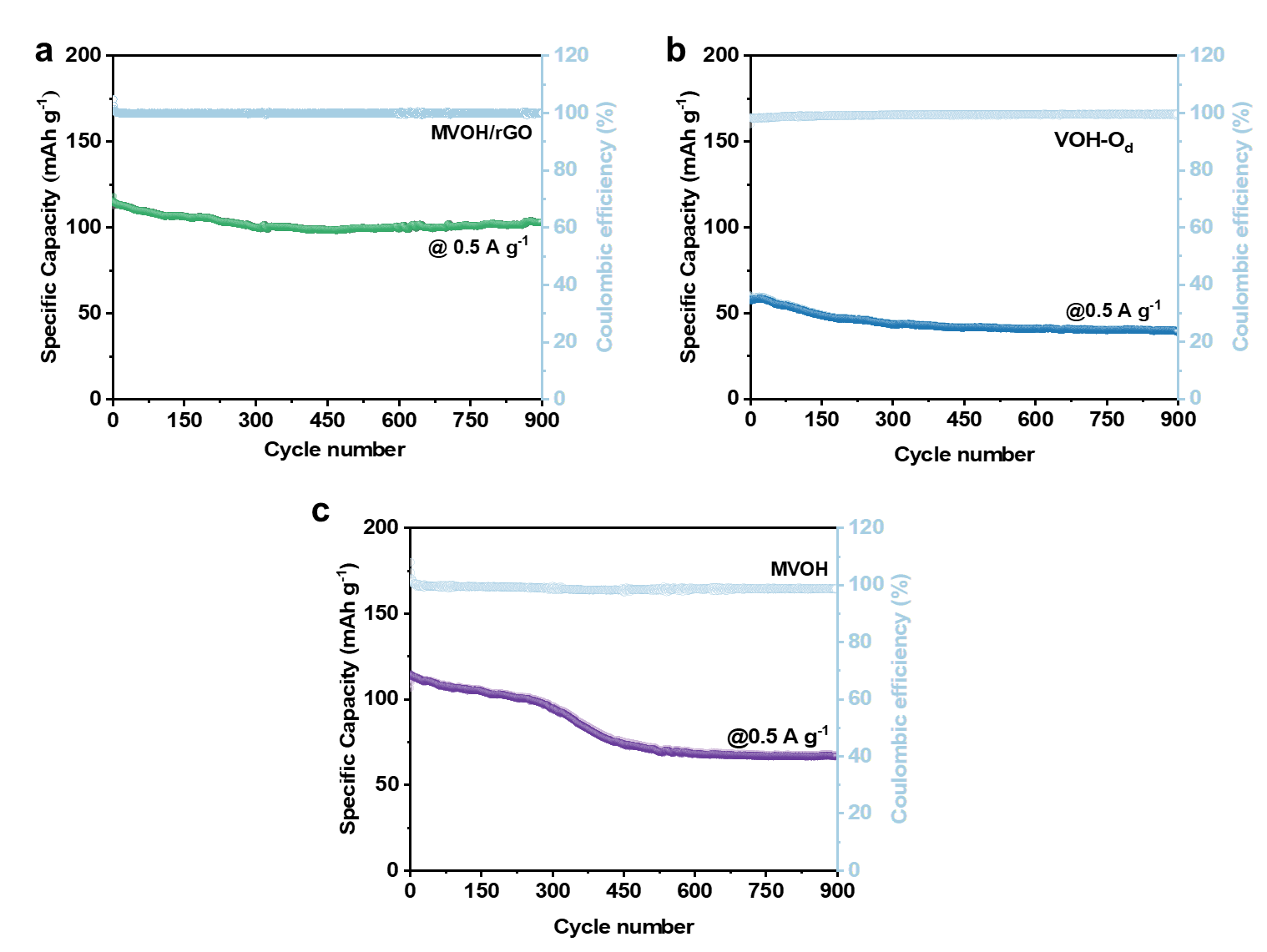
**

**Fig. S14** Long-term cycling performance at 0.5 A g^−1^ of (**a**) MVOH/rGO, (**b**) VOH-O_d_, and (**c**) MVOH cathodes

**
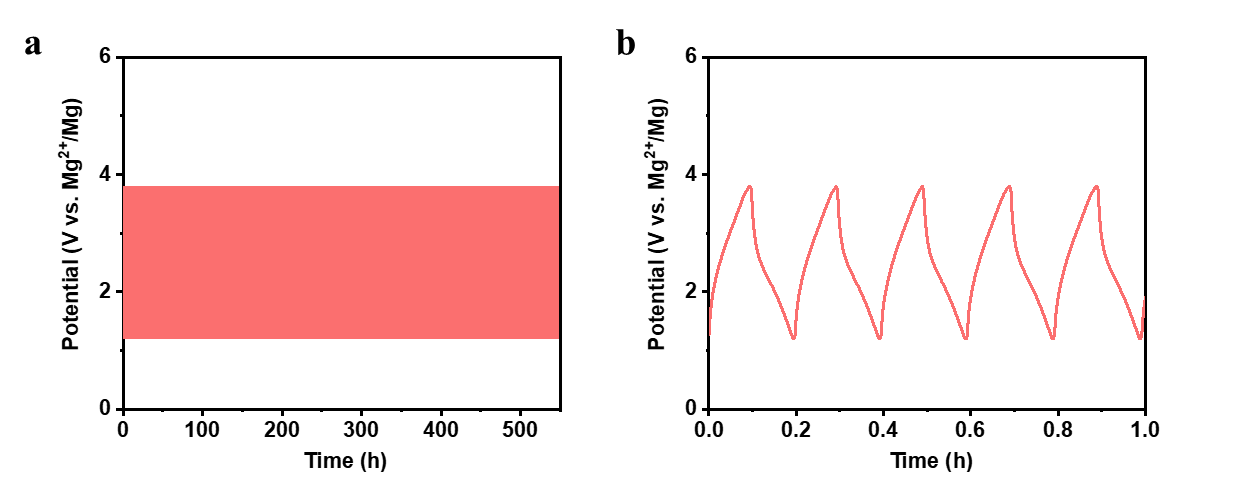
**

**Fig. S15** **(a, b)** Time-potential curves of MVOH/rGO cahode during long-term cycling at 1 A g^−1^

**Fig. S16** Fitting curves for Peaks A−B in CV curves of MVOH/rGO at 0.1−0.8 mV s^−1^

**Fig. S17** Current contribution ratios of capacitive processes for VOH, MVOH, and MVOH/rGO cathodes at 0.1−0.8 mV s^−1^

The contribution ratios of capacitive and diffusion processes in VOH, MVOH, and MVOH/rGO cathodes are calculated from the following equation [S2, S3]:

$\text{i}\text{ = }\text{k}_{\text{1}}\text{v+ }\text{k}_{\text{2}}\text{v}^{\text{1}\text{/2}}$ (S1)

Where ‘*i*’ represents the peak current, and *k_1_* and *k_2_* represent the capacitive and diffusion contribution coefficients, respectively.

**Fig. S18** EIS spectra of VOH, MVOH, and MVOH/rGO cathodes

Mg^2+^ Diffusion coefficient ($\text{D}_{\text{Mg}^{\text{2+}}}$) in the low-frequency region is calculated by the following equation [S4−S7]:

$\text{D}_{\text{Mg}^{\text{2+}}}\text{= }\frac{\text{R}^{\text{2}}\text{T}^{\text{2}}}{\text{2}\text{A}^{\text{2}}\text{n}^{\text{4}}\text{F}^{\text{4}}\text{C}^{\text{2}}\text{σ}_{\text{ω}}^{\text{2}}}$($\text{ω}\text{≫}\frac{\text{2}\text{D}_{\text{Mg}^{\text{2+}}}}{\text{L}^{\text{2}}}$) (S2)

Where R represents the gas constant, T is the absolute temperature, A denotes the electrode area, n stands for the amount of charge transferred per unit of molecule, F is the Faraday constant, C signifies the ion concentration in the electrolyte, and σ_ω_ represents the Warburg coefficient. The low-frequency region σ_ω_ and the real part of impedance (Z') are related by the following equation:

$\text{ Z}^{\text{'}}\text{=}\text{ R}_{\text{s}}\text{ + }\text{R}_{\text{ct}}\text{ +} \text{σ}_{\text{ω}}^{\text{1}\text{/2}}$ (S3)

**
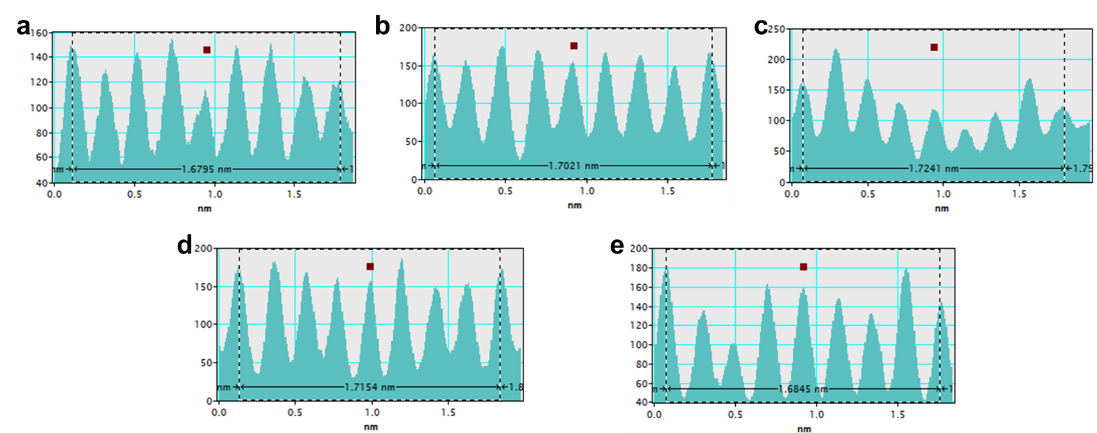
**

**Fig. S19** (**a−e**) Interlayer spacing results of ex-situ TEM characterization


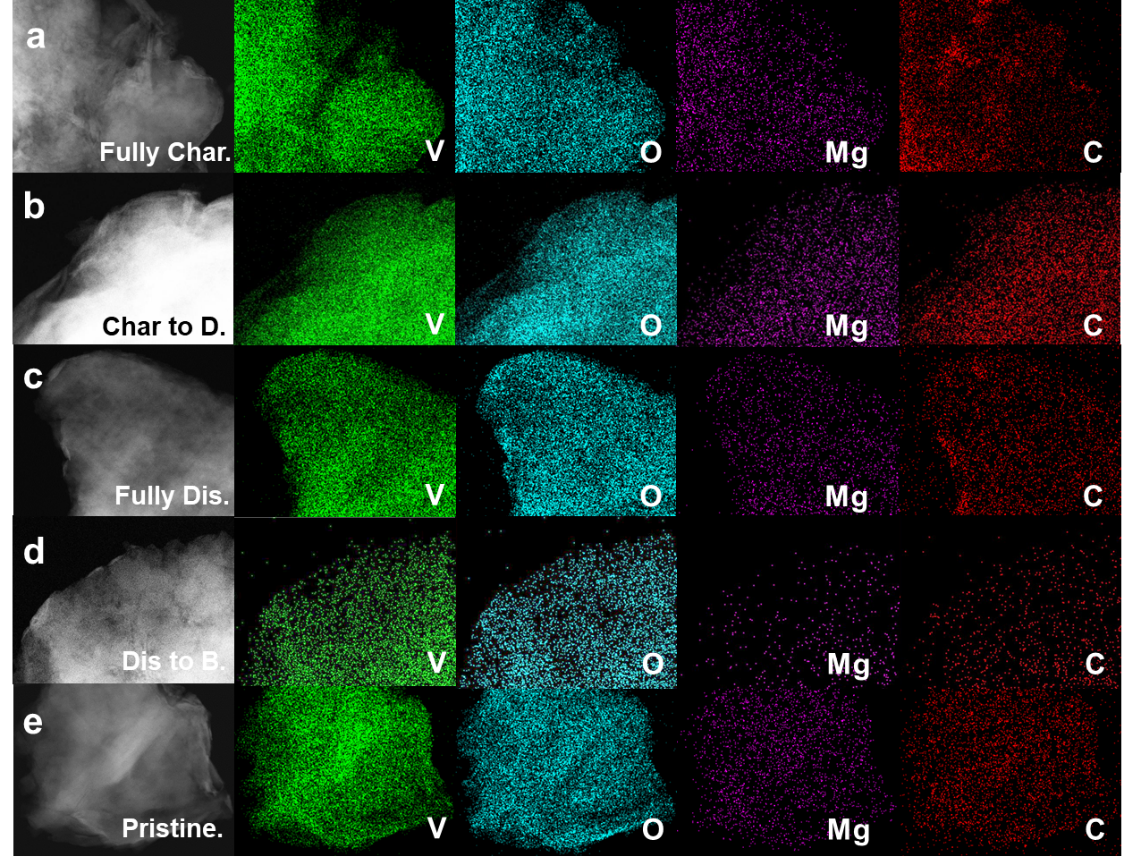


**Fig. S20** Elemental mapping results of V, O, Mg, and C elements in MVOH/rGO at (**a**) fully charged, (**b**) charge states of D, (**c**) fully discharged, (**d**) discharg state of B, and (**e**) pristine states

**Fig. S21** Cycling performance of Mg foil//MVOH/rGO full cell at 0.05 A g^−1^

**Table S1** ICP–OES results of Mg and V elements in MVOH/rGO and MVOH

| Samples | Elements | C_1_(mg/L) | RSD | Mg:V  (atomic ratio) | Formula |
| --- | --- | --- | --- | --- | --- |
| MVOH/rGO-1 | Mg/V | 0.963/58.27 | 0.04%/1.14% | 0.07: 2 | Mg_0.07_V_2_O_5_ |
| MVOH/rGO-2 | Mg/V | 0.959/58.06 | 0.04%/1.14% | 0.07: 2 | Mg_0.07_V_2_O_5_ |
| MVOH-1 | Mg/V | 0.322/28.37 | 0.013%/0.56% | 0.05: 2 | Mg_0.05_V_2_O_5_ |
| MVOH-2 | Mg/V | 0.320/27.87 | 0.013%/0.55% | 0.05: 2 | Mg_0.05_V_2_O_5_ |

**Table S2** XRD Rietveld refinement results of MVOH/rGO

| Samples | R_wp_ | R_p_ | GOF | χ² |
| --- | --- | --- | --- | --- |
| MVOH/rGO | 3.49% | 2.77% | 1.37 | GOF^2 |

Where, R_wp_ represents the Weighted Profile R-factor, R_p_ is the Profile R-factor, GOF denotes the Goodness of Fit, χ² stands for the Chi-squared, respectively.

**Table S3** Comparison of cycling performance between MVOH/rGO and previously reported cathodes

| Cathode | Anode | Electrolyte | Cycling performance | Refs. |
| --- | --- | --- | --- | --- |
| V_2_O_5-x_ | AC | 0.5 M Mg(ClO_4_)_2_ | ~78.2% after 400 cycles at 0.  1 A g^−1^ | [S7] |
| Mn_0.04_V_2_O_5_·1.17H_2_O | AC | 0.3 M Mg(TFSI)_2_ | 82% after 10,000 cycles at 2 A g^−1^ | [S8] |
| Mg_0.3_V_2_O_5_·1.1H_2_O | AC | 0.3 M Mg(TFSI)_2_ | 80% after 10,000 cycles at 2 A g^−1^ | [S9] |
| PANI-V_2_O_5_ | AC | 0.3 M Mg(TFSI)_2_ | ~80% after 500 cycles at 4 A g^−1^ | [S10] |
| SNVO_X_-CNT | AC | 0.5 M Mg(ClO_4_)_2_ | ~82% after 400 cycles at 0.1 A g^−1^ | [S11] |
| V_2_O_5_-PEDOT | AC | 0.3 M Mg(TFSI)_2_ | 67.3% after 500 cycles at 0.5 A g^−1^ | [S6] |
| NaV_2_O_2_(PO_4_)_2_F/rGO | AC | 0.3 M Mg(TFSI)_2_ | 76% after 9,500 cycles at 0.5 A g^−1^ | [S12] |
| (NH_4_)_2_V_6_O_16_·1.5H_2_O | AC | 0.5 M Mg(ClO_4_)_2_ | ~63% after 50 cycles at 0.1 A g^−1^ | [S13] |
| NaV_8_O_20_·nH_2_O | - | TEGDME/H_2_O | ~52.5% after 1,000 cycles at 1.5 A g^−1^ | [S14] |
| PANI/CC | AC | 0.3 M Mg(TFSI)_2_ | 97.3% after 1500 cycles at 1 A g^−1^ | [S15] |
| Mg(Mg_0.5_V_1.5_)O_4_ | AC | 0.3 M Mg(TFSI)_2_ | 71.4% after 500 cycles at 1 A g^−1^ | [S16] |
| MVOH/rGO | AC | 0.5 M Mg(TFSI)_2_ | 95% after 7,000 cycles at 1 A g^−1^ | This work |

**Table S4** Comparison of cycling performance between Mg foil//MVOH/rGO full cell and previously reported RMMBs

| Cathode | Anode | Electrolyte | Cycling performance | Refs. |
| --- | --- | --- | --- | --- |
| Na_2_Ti_6_O_13_ | Mg foil | 0.4 M APC | 58.8% after 300 cycles at 0.1 A g^−1^ | [S17] |
| NiS@C-C | Mg foil | APC-LiCl | 65% after 45 cycles at 0.05 A g^−1^ | [S18] |
| Te@CSs | Mg foil | 0.4 M APC | 77.1% after 500 cycles at 0.5 A g^−1^ | [S19] |
| RFC/V_2_O_5_ | Mg foil | 0.2 M [Mg_2_(μ-Cl)_2_(DME)_4_][AlCl_4_]_2_ in DME | 67% after 100 cycles at 0.32 A g^−1^ | [S20] |
| PA-VOPO_4_ | Mg foil | 0.25 M APC | 70% after 500 cycles at 0.1 A g^−1^ | [S21] |
| PVO | Mg foil | 0.2 M Mg(CF_3_SO_3_)_2_-  MgCl_2_-AlCl_3_ | 69.6% after 50 cycles at 0.1 A g^−1^ | [S10] |
| Ag_2_Se@C | Mg foil | 0.2 M APC | 65% after 500 cycles at 0.2 A g^−1^ | [S22] |
| Ti_3_C_2_Tx@C | Mg AZ31 | 0.4 M Mg_2_Cl_3_^+^  ·AlPh_2_Cl_2_-/THF | 85% after 400 cycles at 0.05 A g^−1^ | [S23] |
| Mo_6_S_8_ | Mg foil | PDEGVE@GF GPE | 80.3% after 500 cycles at 0.13 A g^−1^ | [S24] |
| MoS_2_/graphene | Mg foil | 0.25 M APC | 83% after 500 cycles at 0.5 A g^−1^ | [S25] |
| CuSe | Mg foil | 0.3 M Mg[B(hfip)_4_]_2_/DME-NaOTf | 84.0% after 100 cycles at 0.1 A g^−1^ | [S26] |
| V_2_O_5_-PEDOT | Mg foil | 0.4 M APC-CTAB | ~49.7% after 150 cycles at  0.1 A g^−1^  ~62.3% after 500 cycles at  0.5 A g^−1^ | [S27] |
| NaV_3_O_8_·1.69H_2_O | Mg foil | 0.4 M APC | 80% after 100 cycles at 0.05 A g^−1^ | [S28] |
| MVOH/rGO | Mg foil | 0.4 M APC-CTAB | 84% after 850 cycles at 0.1 A g^−1^ | This work |

**Supplementary References**

[S1] X. Zhao, L. Li, L. Zheng, L. Fan, Y. Yi, et al., 3d‐orbital regulation of transition metal intercalated vanadate as optimized cathodes for calcium‐ion batteries. Adv. Funct. Mater. **34**, 2309753 (2023). <https://doi.org/10.1002/adfm.202309753>

[S2] Y. Zheng, Z. Yao, Z. Shadike, M. Lei, J. Liu, et al., Defect-concentration-mediated T-Nb_2_O_5_ anodes for durable and fast-charging Li-Ion batteries. Adv. Funct. Mater. **32**, 2107060 (2021). <https://doi.org/10.1002/adfm.202107060>

[S3] J. Liu, Y. Wang, C. Yang, K. Mu, S. Wang, et al., A microcapsule‐assistant self‐healing magnesium battery cathodes. Energy Technol. **9**, 2100393 (2021). <https://doi.org/10.1002/ente.202100393>

[S4] Q. Zong, Q. Wang, C. Liu, D. Tao, J. Wang, et al., Potassium ammonium vanadate with rich oxygen vacancies for fast and highly stable Zn-Ion storage. ACS Nano **16**, 4588-4598 (2022). <https://doi.org/10.1021/acsnano.1c11169>

[S5] D. Wu, J. Zeng, H. Hua, J. Wu, Y. Yang, et al., NaV_6_O_15_: A promising cathode material for insertion/extraction of Mg^2+^ with excellent cycling performance. Nano Res. **13**, 335-343 (2020). <https://doi.org/10.1007/s12274-019-2602-6>

[S6] Y. S. Joe, M. S. Kang, G. Jang, S. J. Lee, P. Nakhanivei, et al., Intercalation of bilayered V_2_O_5_ by electronically coupled pedot for greatly improved kinetic performance of magnesium ion battery cathodes. Chem. Eng. J. **460**, 141706 (2023). <https://doi.org/10.1016/j.cej.2023.141706>

[S7] D. Wu, Y. Zhuang, F. Wang, Y. Yang, J. Zeng, et al., High-rate performance magnesium batteries achieved by direct growth of honeycomb-like V_2_O_5_ electrodes with rich oxygen vacancies. Nano Res. **16**, 4880–4887 (2023). <https://doi.org/10.1007/s12274-021-3679-2>

[S8] X. Deng, Y. Xu, Q. An, F. Xiong, S. Tan, et al., Manganese ion pre-intercalated hydrated vanadium oxide as a high-performance cathode for magnesium ion batteries. J. Mater. Chem. A. **7**, 10644–10650 (2019). <https://doi.org/10.1039/c8ta11236c>

[S9] Y. Xu, X. Deng, Q. Li, G. Zhang, F. Xiong, et al., Vanadium oxide pillared by interlayer Mg^2+^ ions and water as ultralong-life cathodes for magnesium-ion batteries. Chem **5**, 1194–1209 (2019). <https://doi.org/10.1016/j.chempr.2019.02.014>

[S10] C. Zuo, Y. Xiao, X. Pan, F. Xiong, W. Zhang, et al., Organic-inorganic superlattices of vanadium oxide@polyaniline for high-performance magnesium-ion batteries. ChemSusChem **14**, 2093–2099 (2021). <https://doi.org/10.1002/cssc.202100263>

[S11] D. Wu, Z. Wen, H. Jiang, H. Li, Y. Zhuang, et al., Ultralong-lifespan magnesium batteries enabled by the synergetic manipulation of oxygen vacancies and electronic conduction. ACS Appl. Mater. Interfaces **13**, 12049-12058 (2021). <https://doi.org/10.1021/acsami.1c00170>

[S12] J. Wang, S. Tan, G. Zhang, Y. Jiang, Y. Yin, et al., Fast and stable Mg^2+^ intercalation in a high voltage NaV_2_O_2_(PO_4_)_2_F/rGO cathode material for magnesium-ion batteries. Sci. China Mater. **63**, 1651–1662 (2020). <https://doi.org/10.1007/s40843-020-1311-1>

[S13] L. Wei, R. Lian, D. Wang, Y. Zhao, D. Yang, et al., Magnesium ion storage properties in a layered (NH_4_)_2_V_6_O_16_·1.5H_2_O nanobelt cathode material activated by lattice water. ACS Appl. Mater. Interfaces **13**, 30625–30632 (2021). <https://doi.org/10.1021/acsami.1c06398>

[S14] X. Wang, X. Zhang, G. Zhao, H. Hong, Z. Tang, et al., Ether-water hybrid electrolyte contributing to excellent Mg ion storage in layered sodium vanadate. ACS Nano **16**, 6093–6102 (2022). <https://doi.org/10.1021/acsnano.1c11590>

[S15] P. Luo, Y. Xiao, J. Yang, C. Zuo, F. Xiong, et al., Polyaniline nanoarrays/carbon cloth as binder-free and flexible cathode for magnesium ion batteries. Chem. Eng. J. **433**, 133772 (2021). <https://doi.org/10.1016/j.cej.2021.133772>

[S16] C. Zuo, W. Tang, B. Lan, F. Xiong, H. Tang, et al., Unexpected discovery of magnesium-vanadium spinel oxide containing extractable Mg^2+^ as a high-capacity cathode material for magnesium ion batteries. Chem. Eng. J. **405**, 127005 (2021). <https://doi.org/10.1016/j.cej.2020.127005>

[S17] L. Luo, Y. Zhen, Y. Lu, K. Zhou, J. Huang, et al., Structural evolution from layered Na_2_Ti_3_O_7_ to Na_2_Ti_6_O_13_ nanowires enabling a highly reversible anode for Mg-ion batteries. Nanoscale **12**, 230-238 (2020). <https://doi.org/10.1039/c9nr08003a>

[S18] G. Zhu, G. Xia, H. Pan, X. Yu. Size-controllable nickel sulfide nanoparticles embedded in carbon nanofibers as high-rate conversion cathodes for hybrid Mg-based battery. Adv. Sci. **9**, e2106107 (2022). <https://doi.org/10.1002/advs.202106107>

[S19] Z. Chen, Q. Yang, D. Wang, A. Chen, X. Li, et al., Tellurium: a high-performance cathode for magnesium ion batteries based on a conversion mechanism. ACS Nano **16**, 5349–5357 (2022). <https://doi.org/10.1021/acsnano.1c07939>

[S20] Y. Cheng, Y. Shao, V. Raju, X. Ji, B. L. Mehdi, et al., Molecular storage of Mg ions with vanadium oxide nanoclusters. Adv. Funct. Mater. **26**, 3446–3453 (2016). <https://doi.org/10.1002/adfm.201505501>

[S21] L. Zhou, Q. Liu, Z. Zhang, K. Zhang, F. Xiong, et al., Interlayer-spacing-regulated VOPO_4_ nanosheets with fast kinetics for high-capacity and durable rechargeable magnesium batteries. Adv. Mater. **30**, e1801984 (2018). <https://doi.org/10.1002/adma.201801984>

[S22] J. H. Ha, B. Lee, J. H. Kim, B. W. Cho, S.-O. Kim, et al., Silver chalco-genides (Ag_2_X, X=S, Se) nanoparticles embedded in carbon matrix for facile magnesium storage via conversion chemistry. Energy Storage Mater. **27**, 459–465 (2020). <https://doi.org/10.1016/j.ensm.2019.12.008>

[S23] F. Liu, Y. Liu, X. Zhao, X. Liu, L.-Z. Fan. Pursuit of a high-capacity and long-life Mg-storage cathode by tailoring sandwich-structured Mxene@carbon nanosphere composites. J. Mater. Chem. A. **7**, 16712–16719 (2019). <https://doi.org/10.1039/c9ta02212k>

[S24] X. Ge, F. Song, A. Du, G. Sun, S. Zhang, et al., Stable anion-rectifying poly (alkoxide magnesium) electrolytes for reversible magnesium metal batteries. ACS Energy Lett. **8**, 3685-3692 (2023). <https://doi.org/10.1021/acsenergylett.3c01192>

[S25] C. Wu, G. Zhao, X. Yu, C. Liu, P. Lyu, et al., MoS_2_/graphene heterostructure with facilitated Mg-diffusion kinetics for high-performance rechargeable magnesium batteries. Chem. Eng. J **412**, 128736 (2021). <https://doi.org/10.1016/j.cej.2021.128736>

[S26] Y. Shen, Y. Wang, Y. Miao, Q. Li, X. Zhao, et al., Anion-incorporated Mg-ion solvation modulation enables fast magnesium storage kinetics of conversion-type cathode materials. Adv. Mater. **35**, e2208289 (2023). <https://doi.org/10.1002/adma.202208289>

[S27] Z. Yao, Y. Yu, Q. Wu, M. Cui, X. Zhou, et al., Maximizing magnesiation capacity of nanowire cluster oxides by conductive macromolecule pillaring and multication intercalation. Small **17**, 2102168 (2021). <https://doi.org/10.1002/smll.202102168>

[S28] M. Rashad, H. Zhang, M. Asif, K. Feng, X. Li, et al., Low-cost room-temperature synthesis of NaV_3_O_8_·1.69H_2_O nanobelts for Mg batteries. ACS Appl. Mater. Interfaces **10**, 4757–4766 (2018). <https://doi.org/10.1021/acsami.7b18682>
